# A Pilot Study of Telmisartan for Visceral Adiposity in HIV Infection: The Metabolic Abnormalities, Telmisartan, and HIV Infection (MATH) Trial

**DOI:** 10.1371/journal.pone.0058135

**Published:** 2013-03-14

**Authors:** Jordan E. Lake, Chi-Hong Tseng, Judith S. Currier

**Affiliations:** 1 Division of Infectious Diseases, Department of Medicine, University of California Los Angeles, Los Angeles, California, United States of America; 2 Division of General Internal Medicine and Health Services Research, Department of Medicine, University of California Los Angeles, Los Angeles, California, United States of America; Rush University, United States of America

## Abstract

**Background:**

Visceral adiposity in the setting of HIV infection and antiretroviral therapy (ART) is not fully understood, and treatment options remain limited. Telmisartan, an angiotensin receptor blocker and partial PPAR-γ agonist, has been shown to decrease visceral fat and improve metabolic and inflammatory parameters in HIV-uninfected subjects.

**Methods:**

HIV-infected subjects with HIV-1 RNA <50 copies/mL on ART and (women/men) waist circumference >94/95 cm or waist: hip ratio >0.88/0.94 received open-label telmisartan 40 mg po daily for 24 weeks. Adipose tissue (AT) volumes were quantified by L4–L5 single slice computed tomography. Metabolic and inflammatory markers were obtained fasting. Thirty-five subjects provided 80% power to detect a 10% 24-week decrease in visceral AT (VAT, two-sided α = 0.05).

**Results:**

Thirty-five subjects enrolled and completed the protocol. At entry (median or %): age 49 years, 43% female, 77% non-white, 91% non-smokers, CD4+ T cell count 590 cells/mm^3^, BMI 31 kg/m^2^. AT responses were heterogeneous, with statistically significant losses of median (IQR) total (TAT, 2.9% (−9.8, 0.7), p = 0.03) and subcutaneous (SAT, −2.7% (−9.8, 1.1), p = 0.03) AT, but not VAT (−2.7% (−20.5, 14.2), p = 0.53). Significant decreases in waist circumference and waist:hip ratio occurred (both p<0.001) without BMI or weight changes. In an exploratory analysis, significant increases in TNF-α occurred among female subjects without changes in other inflammatory or metabolic markers. No related adverse events occurred.

**Conclusions:**

Telmisartan was well tolerated. Small losses of AT from all depots were observed after 24 weeks of telmisartan therapy. Further study is needed to determine whether HIV-infected patients can receive metabolic benefits from telmisartan.

**Trial Registration:**

ClinicalTrials.gov NCT01088295

## Introduction

In the setting of HIV infection, lipohypertrophy is characterized by truncal subcutaneous (SAT) and visceral (VAT) fat accumulation that is often associated with metabolic abnormalities such as hyperlipidemia, insulin resistance, and increased cardiovascular risk [1]–[5]. Currently, treatment options to improve lipohypertrophy and its associated comorbidities in HIV-infected patients are limited.

Metformin, which improves insulin sensitivity in patients with diabetes and polycystic ovarian syndrome, has been shown in HIV infection to improve VAT accumulation but exacerbate peripheral lipoatrophy, [6], [7] which may be an independent risk factor for cardiovascular disease. Both HIV and antiretroviral therapy (ART, specifically the protease inhibitor and nucleoside reverse transcriptase inhibitor classes of agents) may modulate lipodystrophy via down-regulation of partial peroxisome proliferator-activated receptor-gamma (PPAR-γ) [8], [9]. Thiazolidinediones, which also activate PPAR-γ, have not consistently been shown to have an effect on VAT [10] in the setting of HIV infection, and have untoward side effects such as weight gain, fluid retention, and lipid abnormalities. The effects of low-dose growth hormone on VAT are promising, but short term negative effects on insulin sensitivity were observed, lipoatrophy worsened, use requires patients to receive regular subcutaneous injections, and effects appear to diminish quickly when the drug is stopped [11], [12]. Similarly, the growth hormone-releasing factor tesamorelin has been shown to decrease VAT and improve lipids in HIV-infected patients with milder side effects than growth hormone, but it also requires injection, its effects appear to diminish quickly after the drug is stopped, and long-term safety data are lacking [13], [14].

Telmisartan is a renin-angiotensin system (RAS) antagonist and PPAR-γ agonist approved for the treatment of essential hypertension. It has also been shown to decrease VAT volume, total cholesterol, and low-density lipoprotein (LDL) levels, and improve fasting glucose levels, high-density lipoprotein (HDL), and markers of vascular inflammation in HIV-negative patients with the metabolic syndrome [15]–[17]. Specifically, in patients with the metabolic syndrome and newly-diagnosed-hypertension, Shimbakuro and colleagues demonstrated a 10% (approximate) 24-week reduction in VAT that was accompanied by improved insulin sensitivity and glucose tolerance, higher adiponectin levels, and decreased C-reactive protein (CRP) [15]. In hypertensive Japanese patients, Chujo et al. demonstrated a 10% reduction in VAT that was accompanied by increased HDL cholesterol and adiponectin and decreased interleukin-6 (IL-6) [17]. Derosa et al. demonstrated more rapid lipid and glucose benefits with telmisartan vs. irbesartan in Italian diabetic patients on rosiglitazone therapy [16].

Telmisartan may improve adipose tissue (AT) structure and function via multiple mechanisms. First, through RAS inhibition, telmisartan stimulates adipocyte maturation, decreases adipocyte size, improves adipocyte metabolism, and promotes fat deposition into ectopic sites rather than mature adipocytes [18]–[20]. Second, PPAR-γ agonism stimulates fat redistribution from VAT to SAT [21]. Third, VAT accumulation is associated with suppression of adiponectin secretion [22] and increased angiotensinogen [23]. The combined effects of adiponectin depletion and angiotensinogen excess create a link between HIV- and ART-related lipohypertrophy and RAS-induced tissue dysfunction in HIV-infected patients.

The effects of telmisartan on AT in HIV-infected patients have not been studied. Limited clinical data in hypertensive patients suggest HIV-infected patients may receive similar benefits on lipid levels and measures of hepatic insulin sensitivity to those observed in HIV-uninfected populations [24], [25]. Telmisartan has been studied at standard doses in both hypertensive and normotensive patients, and has an excellent safety profile in both populations [26].

In a 24-week, open label, single arm trial, the effects of telmisartan 40 mg po daily on AT volumes and metabolic parameters in HIV-infected men and women well controlled on ART with central adiposity were assessed. The purpose of this study was to obtain pilot data to inform a larger placebo controlled trial. Primary endpoint results of this pilot study are presented here.

## Materials and Methods

### Patient Population

Subjects were recruited between May and October 2010 at the UCLA Clinical AIDS Research and Education (CARE) Center, and followed until April 2011. The protocol for this trial and supporting CONSORT checklist are available as supporting information; see **Checklist S1** and **Protocol S1**. Inclusion criteria included: age ≥18 years; central fat accumulation (defined as (women/men) waist circumference >94/95 cm or waist:hip ratio >0.88/0.94, similar to studies of growth hormone releasing factor [14]); HIV-1 RNA <50 copies/mL on ART at screening and for ≥12 weeks prior to entry; no change in ART for ≥12 weeks prior to entry; systolic blood pressure >115 mmHg; and ability and willingness to provide informed consent.

Exclusion criteria included: pregnancy or breastfeeding; uncontrolled hypertension; current use of thiazolidinediones or other angiotensin receptor blockers (ARBs); current use of nelfinavir or etravirine (due to possible cytochrome P-450 2C19 inhibition by telmisartan); intent to significantly modify diet or exercise habits during the study period; absolute neutrophil count <750 cells/mm^3^, hemoglobin <10 gm/dL, creatinine clearance <30 mL/min, or aspartate aminotransferase (AST) or alanine aminotransferase (ALT) >3 times the upper limit of normal; untreated renal artery stenosis; unstable coronary artery disease, angina, or decompensated congestive heart failure; history of intolerance to any ARB; and need for ongoing potassium supplementation.

Subjects on stable (no change in dose for ≥12 weeks prior to entry) anti-hypertensive medications were permitted to enroll if their prescribing physician approved the addition of telmisartan to their current regimen. To minimize aggravation of renal function, subjects on angiotensin converting enzyme inhibitors (ACEi) were asked not to titrate their ACEi dose for the 24-week study duration. Subjects on stable lipid-lowering or insulin-sensitizing agents were instructed not to titrate the doses of these medications while on study. Female subjects participating in sexual activity and of reproductive potential were required to use contraception until 4 weeks after discontinuation of telmisartan.

### Ethics Statement

All study documents and procedures were approved by the institutional review board at the University of California, Los Angeles, and all subjects provided written informed consent prior to initiation of study procedures. The study was registered at clinicaltrials.gov (NCT 01088295), and details can be found at http://clinicaltrials.gov/show/NCT01088295.

### Study Design

In a single arm, open label design, enrolled subjects received telmisartan 40 mg po daily (with continued ART) for 24 weeks. No preliminary data on the use of telmisartan in HIV-infected subjects was available during study development, and, given the known sequelae of chronic viral infection and ART, it could not be assumed that HIV-infected patients would receive the same metabolic and anti-inflammatory benefits as HIV-uninfected participants. Therefore, a single arm study design was chosen to facilitate the collection of pilot safety data and an estimate of telmisartan’s effect(s) on AT in the setting of HIV infection.

Subjects with symptomatic hypotension or a significant increase in serum creatinine on telmisartan therapy could dose reduce to telmisartan 20 mg daily. Subjects tolerating the dose reduction were allowed to remain on telmisartan 20 mg daily for the study duration and were not re-challenged with telmisartan 40 mg. Subjects unable to tolerate dose-reduced telmisartan were followed on-study off-drug for the study duration. The primary endpoint was 24-week change in percent computed tomography (CT)-quantified VAT.

### Assessments

Adipose tissue volumes [VAT, SAT and total AT (TAT)] were measured via single slice L4–L5 CT scan at Weeks 0 and 24. CT was chosen over MRI (both can accurately measure VAT volume) to maximize accessibility and minimize cost [27]. Scans were performed at the UCLA Ronald Reagan Medical Center, but standardized and read by a blinded reader at the Tufts University Body Composition Center, which is able to interpret AT volumes with <0.1 cm accuracy (personal communication, Justin Wheeler, Tufts University Body Composition Center).

Waist and hip circumferences were performed according to AIDS Clinical Trials Group standards (https://actgnetwork.org/committees/resource/site-management-clinical-care/training-subcommittee) at Weeks 0 and 24. Waist:hip ratio was calculated from these measurements.

Fasting (≥8 hours) glucose and lipoprotein profiles and HIV-1 RNA (assay sensitivity ≤50 copies/mL) were assessed at Weeks 0, 12, and 24. CD4+ T cell counts were measured at Weeks 0 and 24. All other safety evaluations were performed at Weeks 0, 6, 12, 18, and 24, and included complete blood count with differential, chemistry panel including liver enzymes and serum creatinine, and a pregnancy test. All safety labs were performed in real-time according to local standards. Banked serum and plasma samples were collected at Weeks 0, 12, and 24, and were analyzed in the Laboratory for Clinical Biochemistry Research at the University of Vermont. IL-6 was measured by Chemiluminescent Sandwich ELISA (R&D Systems, sensitivity 0.48 pg/mL, inter-assay variability 6.3%–13.6%); high-sensitivity CRP (hs-CRP) by NB™II nephelometer, N Antiserum to Human CRP (Siemens Diagnostics, sensitivity 0.15 µg/mL, inter-assay variability 2.5%–3.8%); adiponectin by Millipore Human Adipokine Panel A multiplex assay (adjusted to ELISA method by R&D systems, R^2^ = 0.894, sensitivity 4.8 ng/mL, inter-assay variability 3.6%–7.8%); and tumor necrosis factor-alpha (TNF-α, sensitivity 0.63 pg/mL, inter-assay variability 6.2%–11.7%), leptin (sensitivity 16.0 pg/mL, inter-assay variability 2.6%–5.4%) and insulin by Millipore Human Adipokine Panel B multiplex assay (insulin adjusted to Roche Elecsys Immunoassay, R^2^ = 0.982, sensitivity 22.2 µU/mL, inter-assay variability 2.2%–9.4%).

Adverse events (AEs) were graded using the Division of AIDS Table for Grading the Severity of Adult and Pediatric AEs (Version 1.0, December 2004). All ≥Grade 3 clinical events and ≥Grade 2 lab abnormalities obligated reporting to the data management center. Pregnancy obligated reporting to the study team, the sponsor, and the Antiretroviral Pregnancy Registry, as well as discontinuation of telmisartan.

### Statistical Analysis

Sample size for this study was informed by studies of growth hormone releasing factor in HIV lipodystrophy, in which the U.S. Food and Drug Administration defined a ≥8% difference in VAT between treatment and placebo groups as clinically significant, assuming an 18.5% standard deviation [14]. Given the single arm design of this study, an estimated sample size of 27 subjects provided 80% power to detect a 10% reduction in VAT (chosen to achieve greater than the defined minimum clinical significance, with the caveat that an 8% within-group decrease may be larger than an 8% between-group difference) over 24 weeks (two-sided α = 0.05). The sample size was increased to 35 subjects to improve power for secondary endpoints and to account for potential loss to follow-up.

Baseline characteristics by sex were compared using the Mann-Whitney U-test for continuous variables and the Fisher’s exact test for categorical variables. Medians and interquartile ranges are reported for continuous variables, and percentages for categorical data.

Comparison of median 24-week change scores for all AT volumes, circumferences, and lab values was performed using the Wilcoxon signed-rank test. Analysis of mean change scores was also performed and produced similar results (data not shown). Correlations between change scores were tested using Spearman’s rank correlation coefficients. The primary analysis for this pilot study was as-treated, excluding subjects who did not remain on telmisartan and/or did not have an observed primary endpoint. A supplemental intent-to-treat analysis and analyses of log-transformed mean values (vs. median) were also performed and produced similar results (data not shown).

Due to the pilot nature of this study, additional secondary analyses were performed stratifying data by sex (male vs. female) and body mass index (BMI; <30 vs. ≥30 kg/m^2^). Odds ratios were calculated to assess baseline predictors of VAT loss or gain during telmisartan therapy. Multivariable modeling was not performed due to the small sample size.

Additionally, the number of subjects experiencing treatment-related AEs and/or discontinuing treatment for any reason was summarized, including the reason(s) for discontinuation.

All statistical tests were two-sided with a significance level of 0.05. Analyses were exploratory, and did not adjust for multiple testing. Data analysis and management was performed using SAS 9.2 (SAS Institute, Inc., Cary, NC, USA) and R software (www.r-project.org).

## Results

### Patient Population

**Figure 1:**
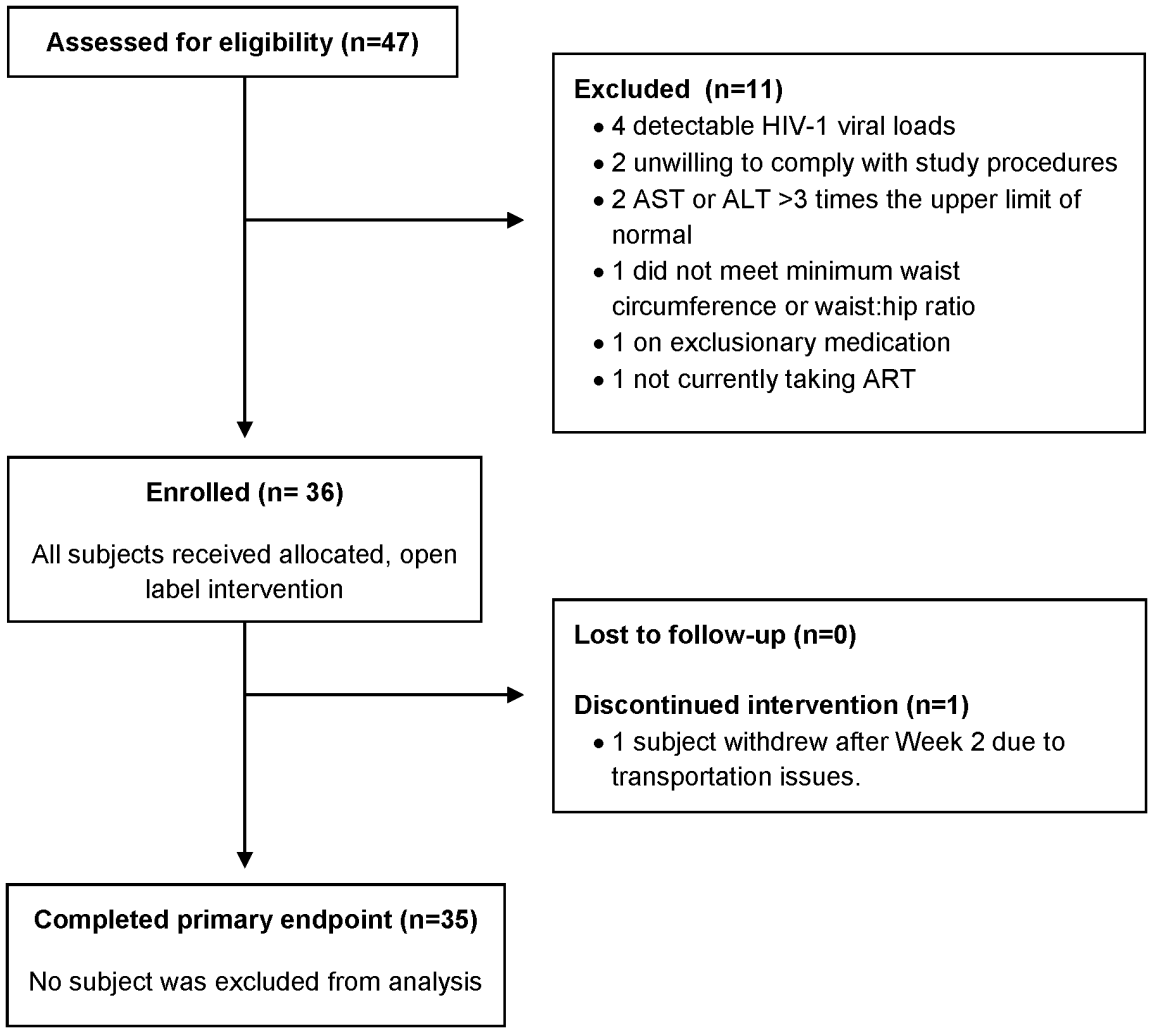
Enrollment and disposition. AST = aspartate aminotransferase, ALT = alanine aminotransferase, ART = antiretroviral therapy.

Forty-seven subjects were screened, 36 enrolled, and 35 completed the Week 24 primary endpoint (**Figure 1**). Reasons for screen failure included: Having a detectable HIV-1 RNA (n = 4), unwillingness to comply with study procedures (n = 2), AST or ALT >3 times the upper limit of normal (n = 2), not meeting minimum waist circumference and/or waist:hip ratio criteria (n = 1), exclusionary concomitant medication (n = 1), and not currently taking ART (n = 1). One subject withdrew for logistical reasons prior to Week 6, and one subject was unable to complete the Week 24 CT scan but was included in the analysis because she completed all other study procedures. Sixteen subjects were unable to have their Weeks 0 and 24 CT scans performed on the same scanner; however, phantom scan comparison revealed no significant scanner discrepancies requiring calculation of a correction factor, and sensitivity analysis revealed AT volumes were precise to ±1 cm^2^ (personal communication, Justin Wheeler). There were no withdrawals due to telmisartan intolerance or AEs, and no subject required de-escalation of telmisartan to the 20 mg dose.

Complete baseline demographic and clinical characteristics are provided in **Table 1**. Thirty-five subjects were included in the as-treated analysis. The median age was 49 years, BMI 31 kg/m^2^, and CD4+ T cell count 590 cells/mm^3^. Nine percent were current smokers, and 75% of subjects self-identified as Black or Hispanic (100% women vs. 60% men). ART at entry included 51% protease inhibitor (PI)-based ART, 20% non-nucleoside reverse transcriptase inhibitor (NNRTI)-based ART, and 26% raltegravir. The most common nucleoside reverse transcriptase inhibitors (NRTIs) were tenofovir (74%) and emtricitabine (66%). Twenty-nine percent of subjects (n = 10) were on stable anti-hypertensive agent at entry, one of whom self-discontinued her ACEi two weeks post-entry. No subject reported a change from baseline in initiation or dosing of lipid- (n = 19) or glucose-lowering agents (n = 5) or androgen supplementation (n = 6) during the 24-week study period. In keeping with a median BMI of 31 kg/m^2^, median AT volumes were large at baseline [VAT 179 cm^2^, SAT 329 cm^2^, TAT 530 cm^2^).

**Table 1:** Demographic and clinical baseline characteristics§.

	Women (n = 15)	Men (n = 20)	Overall (n = 35)
Ethnicity*			
African-American	53%	20%	**34%**
Hispanic	47%	40%	**43%**
White	0%	40%	**23%**
Age (years)	50 (45, 54)	49 (46, 53)	**49 (44, 54)**
BMI (kg/m^2^)	28 (26,38)	31 (28,33)	**31 (27, 34)**
Tobacco Use (Current)	20%	0%	**9%**
CD4 count (cells/mm^3^)	736 (457, 959)	565 (477, 651)	**590 (457, 791)**
PI	47%	55%	**51%**
NNRTI	7%	30%	**20%**
Integrase Inhibitor	27%	25%	**26%**
NRTI Backbone			
Abacavir	20%	15%	**17%**
Lamivudine	40%	10%	**23%**
Emtricitabine	60%	70%	**66%**
Tenofovir	73%	75%	**74%**
VAT (cm^2^)*	113 (97, 156)	221 (178, 245)	**179 (119, 229)**
SAT (cm^2^)*	427 (312, 714)	329 (238, 410)	**375 (250, 430)**
TAT (cm^2^)	532 (451, 836)	530 (469, 598)	**532 (464, 607)**
VAT:SAT*	0.24 (0.19, 0.36)	0.59 (0.45, 0.96)	**0.45 (0.27, 0.74)**
VAT:TAT*	0.19 (0.16, 0.26)	0.37 (0.31, 0.48)	**0.31 (0.21 0.42)**
Waist Circumference (cm)	103 (95, 125)	107 (100, 116)	**104 (99, 116)**
Hip Circumference (cm)	109 (97, 127)	102 (99, 110)	**103 (97, 113)**
Waist-Hip Ratio*	0.96 (0.94, 1.01)	1.05 (1.02, 1.08)	**1.01 (0.96, 1.06)**
Systolic Blood Pressure (mmHg)	121 (118, 149)	131 (125, 142)	**130 (120, 144)**
Diastolic Blood Pressure (mmHg)	79 (69, 80)	80 (78, 85)	**80 (74, 84)**
Glucose (mg/dL)	88 (81, 101)	96 (91, 97)	**95 (87, 99)**
Total Cholesterol (mg/dL)	195 (157, 209)	181 (167, 210)	**184 (163, 210)**
Triglycerides (mg/dL)*	112 (86, 120)	160 (102, 224)	**115 (97, 189)**
LDL (mg/dL)	106 (84, 134)	100 (83,124)	**102 (83, 127)**
HDL (mg/dL)*	52 (29, 67)	41 (26, 72)	**45 (37, 51)**
Diabetes†	7%	10%	**9%**
Hypertension†	20%	45%	**34%**
Hyperlipidemia†	27%	60%	**46%**
Hepatitis B	0%	10%	**6%**
Hepatitis C	13%	5%	**9%**
Menopausal	40%	N/A	**N/A**

§ Percent or median with interquartile range.

* Between-group p≤0.05.

† Defined as self-reported diagnosis or on medication at baseline.

BMI = body mass index; PI = protease inhibitor; NNRTI = non-nucleoside reverse transcriptase inhibitor; NRTI = nucleoside reverse transcriptase inhibitor; VAT = visceral adipose tissue; SAT = subcutaneous adipose tissue; TAT = total adipose tissue; LDL = low-density lipoprotein cholesterol; HDL = high-density lipoprotein cholesterol.

### Adipose Tissue Volume, Weight, and Anthropometric Changes

**Figure 2:**
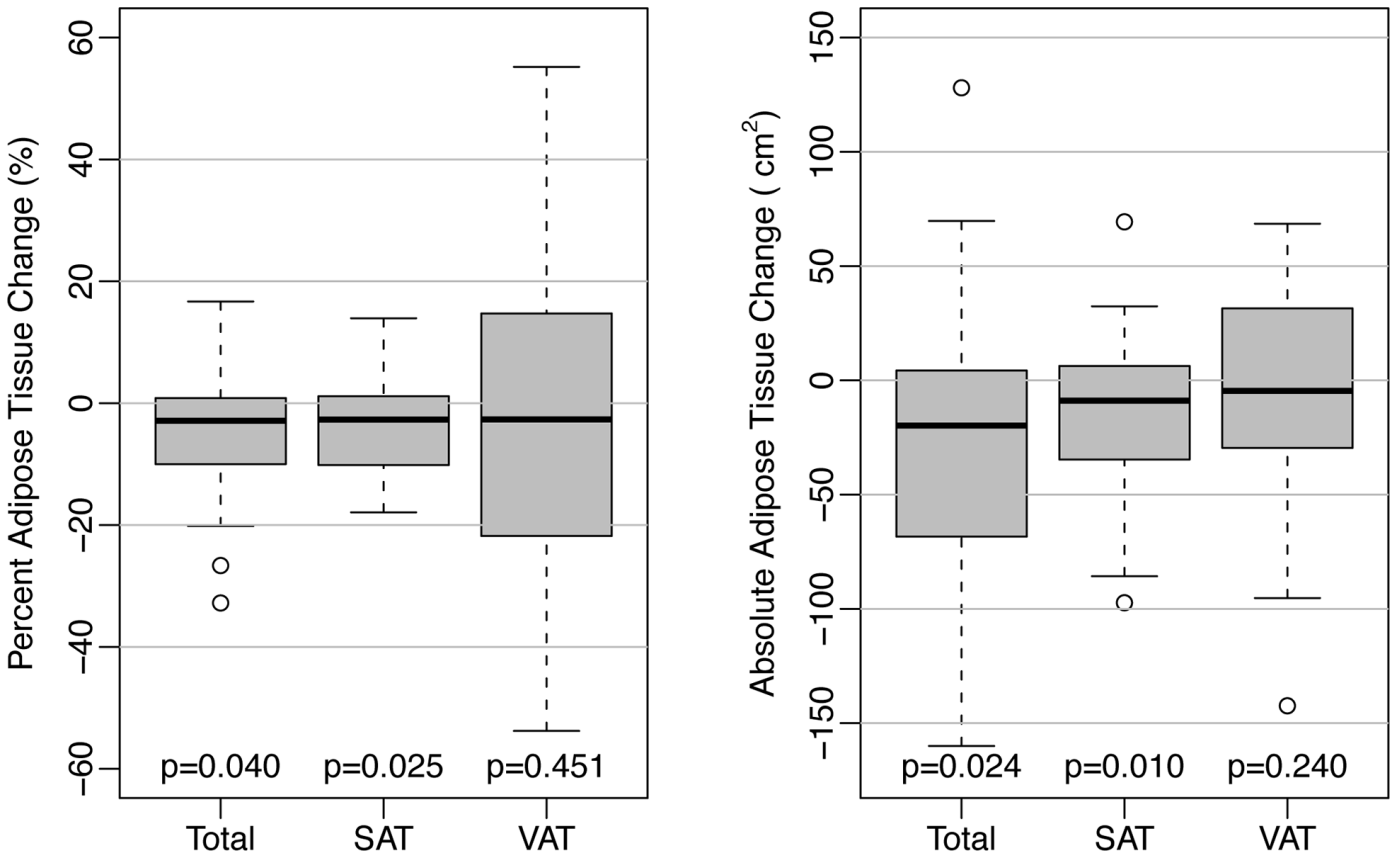
24-week changes in adipose tissue volumes. SAT = subcutaneous adipose tissue, VAT = visceral adipose tissue.

After 24 weeks, changes in AT were heterogeneous. Overall, no statistically significant improvement in median percent VAT (−2.7%; IQR (−20.5, 14.2); p = 0.53) was observed (**Table 2**); however, significant median decreases in TAT (−2.9%; IQR (−9.8, 0.7); p = 0.03) and SAT (−2.7%; IQR (−9.8, 1.1); p = 0.03) did occur (**Figure 2**).

**Table 2:** Overall 24-week changes in clinical and laboratory parameters.

	Median (IQR)	P value
TAT (cm^2^)	−**19.8 (**−**63.3, 4,3)**	**0.03**
% TAT	−**2.9 (**−**9.8, 0.7)**	**0.03**
SAT (cm^2^)	−**8.9 (**−**34.1, 5.7)**	**0.02**
% SAT	−**2.7 (**−**9.8, 1.1)**	**0.03**
VAT (cm^2^)	−4.6 (−29.5, 31.2)	0.41
% VAT	−2.7 (−20.5, 14.2)	0.53
VAT:SAT ratio	0.00 (−0.08, 0.05)	1.00
VAT:TAT ratio	0.00 (−0.03, 0.03)	0.92
Weight (kg)	−0.5 (−2.8, 1.4)	0.36
BMI (kg/m^2^)	−0.2 (−1.0, 0.5)	0.29
Waist (cm)	−**3.3 (**−**5.3, 0.2)**	**<0.001**
Hip (cm)	−0.8 (−2.6, 0.6)	0.09
Waist:hip ratio	−**0.02 (**−**0.04, 0.00)**	**<0.001**
Systolic blood pressure (mmHg)	−**2.0 (**−**15.5, 3.0)**	**0.03**
Diastolic blood pressure (mmHg)	−**6.0 (**−**12.0, 4.5)**	**0.02**
Total cholesterol (mg/dL)	−4.0 (−30.5, 13.5)	0.36
HDL cholesterol (mg/dL)	−0.6 (−5.5, 3.3)	0.28
LDL cholesterol (mg/dL)	0.5 (−16.7, 21.3)	0.99
Triglycerides (mg/dL)	−10.0 (−31.5, 33.0)	0.81
Glucose (mg/dL)	0.0 (−3.0, 4.0)	0.64
Insulin (µU/mL)	2.5 (−4.2, 4.8)	0.42
HOMA−IR	0.5 (−0.8, 1.2)	0.33
Adiponectin (ng/mL)	178.3 (−657.0, 803.8)	0.75
Leptin (pg/mL)	−480.3 (−4498.5, 4316.1)	0.90
CRP (µg/mL)	0.2 (−0.3, 1.0)	0.19
IL-6 (pg/mL)	0.0 (−0.5, 0.6)	0.70
TNF-α (pg/mL)	**0.3 (**−**0.2, 0.8)**	**0.04**

Decreases in median waist circumference (−3.3 cm; IQR (−5.3, 0.2), p<0.001) and waist:hip ratio (−0.02; IQR (−0.04, 0.00); p<0.001) were observed after 24 weeks, without significant changes in hip circumference (−0.8 cm, IQR (−2.7, 0.6), p = 0.09), VAT:TAT ratio (0.00, IQR (−0.03, 0.03), p = 0.92), weight (−0.5 kg, IQR (−2.8, 1.4), p = 0.36), or BMI (−0.2 kg/m^2^, IQR (−1.0, 0.5), p = 0.29).

Overall, changes in BMI and weight correlated highly with changes in TAT (both p≤0.001), SAT (both p≤0.001), and VAT (BMI p = 0.05, weight p = 0.02). Changes in waist circumference correlated with changes in TAT (p = 0.04), with a trend seen for VAT (p = 0.09).

A heterogeneous AT response to telmisartan was observed with some individuals experiencing large losses and others experiencing potentially clinically significant gains (Results of changes in subgroups of patients are summarized in **Table S1**). Fifty-nine percent of subjects lost a median 16% VAT over 24 weeks (p<0.0001). However, the median VAT gain was also 16% (within-group p≤0.01). Subjects losing VAT had significant median declines in TAT (−8.7%, p<0.0001) and SAT (−2.7%, p = 0.04), whereas subjects gaining VAT did not (TAT: 4.4%, p = 0.06; SAT: −1.4%, p = 0.46). Similarly, only subjects losing VAT saw significant improvements in median weight, BMI, waist circumference, and waist:hip ratio (data not shown). The between-group difference in TAT (13.1%) but not SAT (1.3%) change was statistically significant (TAT p<0.0001, SAT p = 0.52). An exploratory univariable analysis was performed to determine baseline predictors of clinically significant changes in VAT (defined as gain or loss >10%) but did not provide additional insight (data not shown), likely due to the small sample size in our study.

**Figure 3:**
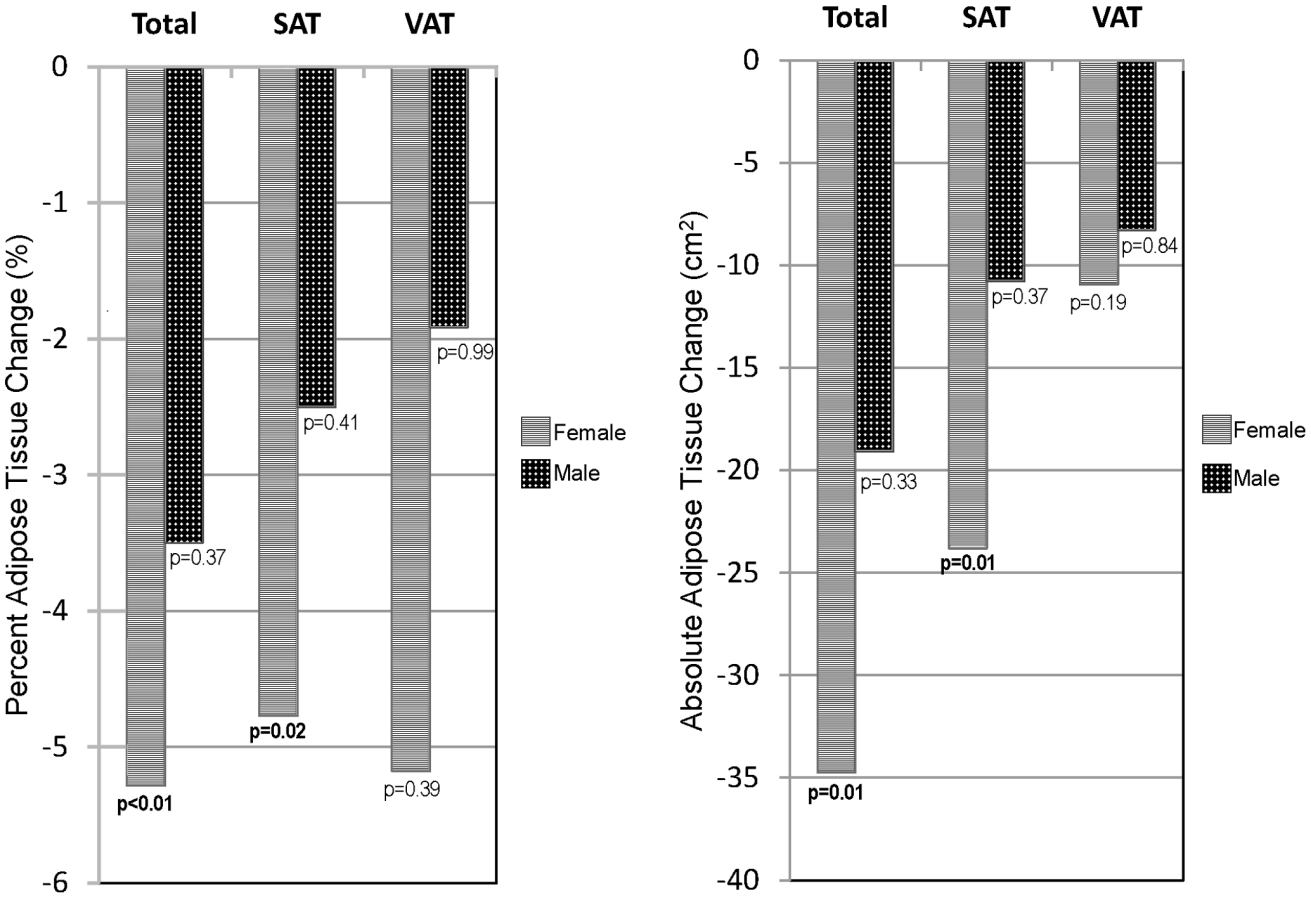
24-week changes in adipose tissue volumes by sex. SAT = subcutaneous adipose tissue, VAT = visceral adipose tissue.

Although this study was not powered to analyze differences by sex, women objectively lost more median VAT and SAT than men (VAT: −5.3% (p = 0.39) vs. −1.2% (p = 0.99), between-group p = 0.50; SAT: −3.3% (p = 0.02) vs. 0.3% (p = 0.41), between-group p = 0.40), and the decrement in TAT was only significant in women (women: −2.5% (p<0.01), men: −3.7% (p = 0.37), between-group p = 0.64, **Figure 3**).

Women also had significantly greater median decrements in waist circumference compared to men (−4.9 cm (p<0.01) vs. −2.2 cm (p = 0.05), between-group p = 0.02), without differences in hip circumference. Accordingly, a trend toward greater waist:hip ratio decline was seen in women. No sex differences in median weight or BMI change were observed (data not shown).

Fifty-one percent of our cohort had a BMI>30 kg/m^2^. Because telmisartan’s metabolic benefits have been described in primarily non-obese subjects, a subgroup analysis of subjects stratified by baseline BMI ≥30 vs. <30 was performed. Subjects with BMI ≥30 tended to lose AT (VAT: −4.0%, p = 0.17; SAT: −4.4%, p = 0.02; TAT: −4.4%, p = 0.02), and subjects with BMI <30 tended to experience AT gains or stabilization (VAT: 8.8%, p = 0.90; SAT: 0.3%, p = 0.74; TAT: −1.7%, p = 0.46), although this difference in trends was not statistically significant. Both groups experienced similar decreases in waist circumference, hip circumference, and waist:hip ratio. Similarly, non-significant changes in median weight and BMI were observed in both groups (data not shown).

### Lipid and Glucose Metabolism

No significant changes in lipids (total cholesterol, HDL cholesterol, LDL cholesterol, or triglycerides), glucose, insulin, or the homeostasis model assessment of insulin resistance (HOMA-IR) were observed overall (**Table 2**) or in any sub-group 24 weeks after initiating telmisartan therapy.

### Inflammatory Biomarkers

In an exploratory analysis, changes in inflammatory biomarker and adipokine levels were assessed (**Table 2** **)**. A median 0.3 pg/mL increase in TNF-α was observed (p = 0.04), with greater increases seen in women (women: 0.6 pg/mL, p = 0.002; men −0.1 pg/mL, p = 0.76; between-group p = 0.06) and subjects losing VAT (VAT loss: 0.4 pg/mL, p = 0.06; VAT gain: 0.1 pg/mL, p = 0.39; between-group p = 0.44; subgroup results provided in **Table S1**). No significant correlations between changes in TNF-α and changes in other clinical or laboratory parameters were observed, although a trend was seen for BMI (r = −0.30, p = 0.08), weight (r = −0.28, p = 0.10), and waist circumference (r = −0.30, p = 0.08).

No significant median change in hs-CRP (0.2 µg/mL, p = 0.19) was demonstrated; however, the majority of subjects (89%) had hs-CRP<5 µg/mL at baseline. No significant changes in IL-6, adiponectin, or leptin occurred overall or in subgroup analyses.

### Safety

No AEs of any grade were related or possibly related to telmisartan therapy. The mean decrement in systolic/diastolic blood pressure over 24 weeks was 7/5 mmHg (median 2/6 mmHg), similar to that seen in HIV-negative subjects. No deaths, major clinical events, or virologic failures occurred in either group.

## Discussion

After 24 weeks, no statistically significant change in VAT was observed with telmisartan therapy, although the response was heterogeneous. It must be acknowledged that the sample size calculation for this trial was based upon a standard deviation (SD) for the change in VAT of 18.5% (as per FDA guidelines) [14]. Our observed SD for percent change in VAT was 24.9%, making it likely that our study was underpowered to observe a change in the primary endpoint. This is supported by the fact that both percent TAT and SAT change reached statistical significance in our analysis; similar absolute percent change values were observed for TAT and SAT, but the SDs for these volumes were much smaller (TAT: 11.5%, SAT: 8.5%). However, it is noteworthy that the large VAT loss observed in some subjects is on par with both the rate and magnitude of response to the growth hormone releasing factor tesamorelin (telmisartan: 59% responders, −20% mean (−16% median) change in VAT over 24 weeks; tesamorelin 68.8% responders, −27.4% mean change in VAT over 26 weeks) [28]. Also important is the fact that statistically significant losses of TAT and SAT have not previously been reported in HIV-uninfected subjects, [15], [17], [29], [30] and are in contrast to the small gains in subcutaneous trunk and limb AT observed with tesamorelin therapy.

Finally, telmisartan was safe and well-tolerated. The observed changes in AT on telmisartan therapy combined with telmisartan’s safety profile and oral formulation suggest that telmisartan may be an effective, tolerable therapeutic option for some HIV-infected patients living with central adiposity.

Several confounding factors may have contributed to inconsistencies between our findings and data previously published in HIV-uninfected populations. First, other studies reporting VAT loss with telmisartan have represented primarily non-obese, Asian patient populations [15], [17]. With a median BMI of 31 kg/m^2^, morbid obesity (and its associated metabolic and inflammatory complications) may have rendered our subjects unsuitable for VAT modification with telmisartan. It is also possible that a chronic stimulus unrelated to the RAS and PPAR-γ systems was overriding any benefit of telmisartan therapy. For example, it has been hypothesized that PIs activate the RAS, [31] and 40 mg of telmisartan daily may not have provided optimal RAS blockade in our 51% of subjects on a PI. Similarly, Blacks and women have lower plasma renin activity than whites and men, respectively [32]. Although we did not measure plasma renin levels (or other RAS axis hormones), sex and race differences could partially account for the observed heterogeneous response to telmisartan. Additionally, angiotensin II type I (AT1) receptor autoantibody production has been reported in renal, hypertensive, and autoimmune disorders. These autoantibodies are pro-inflammatory, and could attenuate the potential metabolic benefits of telmisartan [33]–[35]. It is currently unknown whether subjects in our study produced AT1 receptor autoantibodies.

Our study also explored the relationship(s) between changes in AT and biomarkers of inflammation associated with clinical outcomes, including: CRP and IL-6, which have been associated with mortality in HIV infection;[36]–[39] TNF-α, which is produced, along with IL-6, in excess by lipodystrophic AT; [40], [41] the anti-inflammatory cytokine adiponectin, whose production is suppressed in the setting of VAT accumulation; [42] and leptin, whose secretion increases with AT volume [42]. Although a trend toward decreased IL-6 was seen in subjects losing VAT, some of the changes in inflammatory markers in this study were unexpected (such as the observed overall increase in TNF-α). Multiple considerations must be given to this finding. First, the increase in TNF-α occurred only in women, but sample size limits the generalizability of this finding. Second, the variability of this assay was quite high (see Assessments, above). Third, because there is limited longitudinal data on changes in these markers among HIV-infected patients with lipohypertrophy or obesity, it is possible that a placebo group would have experienced similar directionality but a greater magnitude of change in TNF-α. It is also possible that telmisartan has opposite effects on inflammation in the setting of HIV infection, and/or that the changes we observed were real and beneficial. For example, associations between lower TNF-α production and both all-cause mortality in advanced HIV-infection [43] and the development of the immune reconstitution inflammatory syndrome have been described, [44] suggesting that clinical situations exist where increases in pro-inflammatory cytokines are beneficial or represent a “return to health” phenomenon. Finally, due to the exploratory nature of these biomarker analyses, it is possible that the statistical significance of the observed change in TNF-α represents a type I error. Because all of these scenarios are feasible, a randomized controlled study is needed to further define the effects of telmisartan on inflammation in HIV-infected patients well controlled on ART.

### Limitations

This study has several limitations. First, the single arm, open label design prohibited comparison of the effects of telmisartan to the natural history of disease in this group of subjects with well-controlled HIV infection and central adiposity. Second, the 24-week follow-up period may have been too short to see the desired changes in VAT (although studies of telmisartan in HIV-uninfected patients and studies of tesamorelin demonstrated benefit within a similar time frame). Third, the waist circumference and waist:hip ratio entry criteria failed to distinguish between subjects with isolated, ART-associated lipohypertrophy and generalized obesity, with enrolled subjects having either or both of these types of central adiposity. While the severity of obesity in this study is representative of the population of HIV-infected patients we serve, the presence of mixed HIV lipohypertrophy and generalized obesity is a significant confounder. Future studies of the effects of telmisartan on lipohypertrophy should consider: 1) setting BMI entry criteria to exclude morbidly obese subjects, and, given the observed loss of abdominal SAT in this study, 2) including objective measurements to determine whether peripheral lipoatrophy is a potential untoward effect of telmisartan in the setting of HIV infection.

Fourth, small sample size limited our ability both to identify the HIV-infected patient population most likely to benefit from telmisartan and to determine whether differences exist between subgroups of subjects, including differences by sex. While some differences by sex were observed, larger studies are needed to determine whether metabolic responses to telmisartan vary by sex. Similarly, the combination of no placebo group, small sample size, and obesity in our cohort limited our ability to perform multivariate analysis and interpret secondary endpoints such as changes in inflammatory biomarkers and subgroup analyses. Finally, protocol-defined pill counts were not performed as an objective measure of treatment adherence. However, the mean decrement in blood pressure was similar to other published studies of telmisartan, suggesting reasonable adherence.

Despite these limitations, the overall loss of TAT and SAT and the VAT response rate and magnitude of loss within a subset of participants obligates further study to determine whether HIV-infected patients can receive metabolic benefits from telmisartan therapy.

## Conclusions

Telmisartan was safe and well tolerated in this cohort of virologically suppressed, HIV-infected men and women with central adiposity. Small declines in VAT, TAT, and SAT were observed over 24 weeks, although the loss of VAT did not reach statistical significance. However, in the majority of subjects losing VAT, the magnitude of loss approached that seen with tesamorelin [28]. Further study is needed to better define the effects of telmisartan on AT and inflammatory and metabolic parameters in HIV-infected patients with central fat accumulation.

## Supporting Information

Table S1 **Stratified 24-week changes in clinical and laboratory parameters.**
(DOC)

Protocol S1 **MATH Trial Protocol.**
(PDF)

Checklist S1 **CONSORT Checklist.**
(DOC)
