# Supplementary material for: A Pilot Study of Telmisartan for Visceral Adiposity in HIV Infection: The Metabolic Abnormalities, Telmisartan, and HIV Infection (MATH) Trial
Source: PLoS One. 2013 Mar 14;8(3):e58135. doi: 10.1371/journal.pone.0058135 (PMC3597631; doi:10.1371/journal.pone.0058135)
Supplement: Table S1 — Stratified 24-week changes in clinical and laboratory parameters. (DOC) [file pone.0058135.s001.doc]

**Table S1.** Stratified 24-week changes in clinical and laboratory parameters.

|  | **VAT loss (p)** | **VAT gain (p)** | **Between-VAT group p value** | **Female (p)** | **Male (p)** | **Between-sex p value** | **BMI≥30 (p)** | **BMI<30 (p)** | **Between**  **-BMI group p value** |
| --- | --- | --- | --- | --- | --- | --- | --- | --- | --- |
| TAT (cm^2^) | **-46.2**  **(<0.0001)** | **18.8**  **(0.05)** | **<0.0001** | **-17.2**  **(0.01)** | -23.3  (0.33) | 0.53 | **-26.9**  **(0.02)** | -6.0  (0.50) | 0.25 |
| % TAT | **-8.7**  **(<0.0001)** | 4.4  (0.06) | **<0.0001** | **-2.5**  **(<0.01)** | -3.7  (0.37) | 0.64 | **-4.4**  **(0.02)** | -1.7  (0.46) | 0.42 |
| SAT (cm^2^) | **-14.7**  **(0.02)** | -4.4  (0.46) | 0.34 | **-14.7**  **(0.01**) | 0.5  (0.37) | 0.27 | **-24.4**  **(0.02)** | 0.5  (0.50) | 0.13 |
| % SAT | **-2.7**  **(0.04)** | -1.4  (0.46) | 0.52 | **-3.3**  **(0.02)** | 0.3  (0.41) | 0.40 | **-4.4**  **(0.02)** | 0.3  (0.74) | 0.12 |
| VAT (cm^2^) | **-27.1**  **(0.0001)** | **32.3**  **(<0.0001)** | **<0.0001** | -7.5  (0.19) | -1.3  (0.84) | 0.64 | -7.5  (0.26) | 7.8  (0.63) | 0.65 |
| % VAT | **-16.0**  **(<0.0001)** | **16.4**  **(0.0001)** | **<0.0001** | -5.3  (0.39) | -1.2  (0.99) | 0.50 | -4.0  (0.17) | 8.8  (0.90) | 0.53 |
| VAT:TAT ratio | **-0.02**  **(<0.001)** | **0.03**  **(0.0001)** | **<0.0001** | -0.01  (0.58) | 0.01  (0.78) | 0.64 | 0.00  (0.97) | 0.01  (1.00) | 0.80 |
| Weight (kg) | -**1.8**  (**0.02**) | 0.7  (0.41) | 0.06 | -0.5  (0.13) | -0.7  (1.00) | 0.87 | -2.0  (0.20) | 0.0  (0.66) | 0.15 |
| BMI (kg/m^2^) | **-0.6**  **(0.03)** | 0.3  (0.56) | 0.10 | -0.2  (0.16) | -0.2  (0.97) | 0.47 | -0.8  (0.20) | 0.0  (0.80) | 0.18 |
| Waist circumference (cm) | **-3.9**  **(<0.001)** | -1.7  (0.15) | 0.10 | **-4.9**  **(<0.01)** | **-2.2**  **(0.05)** | **0.02** | **-3.0**  **(0.02)** | **-4.0**  **(<0.01)** | 0.48 |
| Hip circumference (cm) | -1.0  (0.26) | -0.4  (0.23) | 0.96 | -1.0  (0.28) | -0.5  (0.22) | 0.61 | -0.5  (0.97) | **-0.8**  **(0.02)** | 0.16 |
| Waist:hip ratio | **-0.03**  **(<0.001)** | -0.01  (0.43) | 0.13 | **-0.04**  **(<0.01)** | -0.01  (0.06) | 0.06 | **-0.02** (**<0.0001**) | -0.02  (0.09) | 0.55 |
| Systolic blood pressure (mmHg) | -2.0  (0.10) | -2.0  (0.21) | 0.93 | -4.0  (0.11) | -1.5  (0.15) | 0.76 | -2.0  (0.06) | -4.0  (0.24) | 0.62 |
| Diastolic blood pressure (mmHg) | -9.5  (0.15) | **-5.5**  **(0.03)** | 0.75 | -7.0  (0.29) | **-5.5**  **(0.03)** | 0.92 | -5.5  (0.24) | **-7.0**  **(0.03)** | 0.40 |
| Total cholesterol (mg/dL) | -5.5  (0.23 | 1.5  (0.88) | 0.34 | -1.0  (1.00) | -8.5  (0.28) | 0.35 | -4.5  (0.88) | -4.0  (0.25) | 0.62 |
| HDL cholesterol (mg/dL) | -0.6  (0.34) | -0.7  (0.90) | 0.40 | -0.6  (0.45) | -1.3  (0.45) | 0.96 | 0.0  (0.64) | -1.9  (0.24) | 0.77 |
| LDL cholesterol (mg/dL) | -4.1  (0.55) | 2.3  (0.50) | 0.34 | -0.2  (0.89) | 0.6  (0.93) | 1.00 | 1.7  (0.38) | -7.9  (0.43) | 0.20 |
| Triglycerides (mg/dL) | 12.0  (0.60) | -19.0  (0.10) | 0.18 | -8.0  (0.89) | -10.5  (0.70) | 0.76 | -10.5  (0.64) | 8.0  (1.00) | 0.50 |
| Glucose  (mg/dL) | 0.0  (0.78) | 1.5  (0.15) | 0.27 | 0.0  (0.70) | 1.5  (0.25) | 0.36 | 1.0  (0.39) | 0.0  (1.00) | 0.54 |
| Insulin (μU/mL) | 1.8  (0.93) | 2.1  (0.24) | 0.46 | 1.6  (0.56) | 2.6  (0.45) | 0.81 | 1.3  (0.55) | 2.6  (0.49) | 0.99 |
| HOMA-IR | 0.4  (0.90) | 0.5  (0.27) | 0.57 | 0.4  (0.42) | 0.7  (0.50) | 0.81 | 0.3  (0.50) | 0.5  (0.52) | 0.99 |
| Adiponectin (ng/mL) | 25.7  (0.65) | 196.1  (0.50) | 0.96 | -365.8 (0.89) | 196.1  (0.55) | 0.54 | -228.9  (0.53) | 426.5  (0.40) | 0.23 |
| Leptin (pg/mL) | -696.8  (0.57) | 1778.9  (0.50) | 0.44 | -1081.2  (0.80) | -296.0  (0.96) | 0.71 | -1368.2  (0.32) | 2339.6  (0.35) | 0.21 |
| CRP (μg/mL) | 0.2  (0.31) | -0.1  (0.73) | 0.62 | 0.3  (0.12) | 0.0  (0.76) | 0.28 | 0.2  (0.78) | 0.1  (0.16) | 0.55 |
| IL-6 (pg/mL) | -0.2  (0.24) | 0.4  (0.07) | **0.03** | 0.2  (0.45) | -0.0  (0.90) | 0.49 | 0.1  (0.64) | 0.0  (0.31) | 0.34 |
| TNF-α (pg/mL) | 0.4  (0.06) | 0.1  (0.39) | 0.44 | **0.6**  (**0.002**) | -0.1  (0.76) | 0.06 | 0.1  (0.3) | 0.5  (0.1) | 0.60 |
