# Supplementary material for: A Pilot Study of Telmisartan for Visceral Adiposity in HIV Infection: The Metabolic Abnormalities, Telmisartan, and HIV Infection (MATH) Trial
Source: PLoS One. 2013 Mar 14;8(3):e58135. doi: 10.1371/journal.pone.0058135 (PMC3597631; doi:10.1371/journal.pone.0058135)
Supplement: Protocol S1 — MATH Trial Protocol. (PDF) [file pone.0058135.s002.pdf]

**Study Title:**

# Metabolic Abnormalities, Telmisartan, and HIV Infection (MATH) Trial

Version 1.0 dated 10/30/09

Pharmaceutical Support and Study Drug Provided by:

Study Drug:

Micardis® (Telmisartan)

Protocol Co-Chairs:

Jordan E. Lake, M.D.

Judith Currier, M.D.

**TABLE OF CONTENTS**

**PROTOCOL SIGNATURE FORM** .......... **4**

**APPENDICES** .......... **5**

**PROTOCOL ROSTER** .......... ERROR! BOOKMARK NOT DEFINED.

**1.0 STUDY HYPOTHESIS AND OBJECTIVES:** .......... **8**

1.1 HYPOTHESIS .......... 8
1.2 PRIMARY OBJECTIVES: .......... 8
1.3 SECONDARY OBJECTIVES: .......... 8

**2.0 INTRODUCTION** .......... **9**

2.1 BACKGROUND AND RATIONALE .......... 9

**3.0 STUDY DESIGN** .......... **11**

**4.0 SELECTION AND ENROLLMENT OF SUBJECTS** .......... **12**

4.1 INCLUSION CRITERIA .......... 12
4.2 EXCLUSION CRITERIA .......... 12

**5.0 STUDY TREATMENT** .......... **14**

5.1 REGIMENS, ADMINISTRATION, AND DURATION .......... 14
5.2 STUDY PRODUCT FORMULATION AND PREPARATION .......... 14
5.3 PHARMACY: PRODUCT ACQUISITION, DISTRIBUTION, AND ACCOUNTABILITY .......... 14
5.4 CONCOMITANT MEDICATIONS .......... 14

**6.0 CLINICAL EVALUATIONS** .......... **15**

**6.1 SCHEDULE OF EVENTS** .......... **15**

6.2 TIMING OF STUDY EVALUATIONS .......... 16
6.5 CLINICAL ASSESSMENTS .......... 17
6.6 LABORATORY EVALUATIONS .......... 19

**7.0 TOXICITY MANAGEMENT** .......... **20**

7.1. GRADE 1 OR 2 TOXICITY .......... 20
7. 2 GRADE 3 TOXICITY .......... 20
7.3 GRADE 4 TOXICITY .......... 21

**8.0 CRITERIA FOR DISCONTINUATION** .......... **21**

8.1 PERMANENT STUDY DRUG DISCONTINUATION .......... 21
8.2 PREMATURE STUDY DISCONTINUATION .......... 21
8.3 EARLY DISCONTINUATION .......... 22

**9.0 SAMPLE SIZE AND ENDPOINTS** .......... **22**

9.1 SAMPLE SIZE .......... 22
9.2 RANDOMIZATION AND STRATIFICATION .......... 22
9.3 PRIMARY ENDPOINT ASSESSMENTS .......... 22
9.4 SECONDARY ENDPOINTS ASSESSMENTS .......... 22
9.5 STUDY MONITORING .......... 23
9.6 ANALYSES .......... 23

**10.0 DATA COLLECTION AND MONITORING AND ADVERSE EVENT REPORTING** ........ **23**
10.1 Records to Be Kept ........ 23
10.2 Role of Data Management ........ 24
10.3 Clinical Site Monitoring and Record Availability ........ 24
**10.4 SERIOUS ADVERSE EVENT REPORTING** ........ **24**
**11.0 HUMAN SUBJECTS** ........ **25**
11.1Institutional Review Board (IRB) Review and Informed Consent ........ 25
11.2 Subject Confidentiality ........ 25
11.3 Study Discontinuation ........ 25
**12.0 PUBLICATION OF RESEARCH FINDINGS** ........ **25**
**13.0 BIOHAZARD CONTAINMENT** ........ **25**
**14.0 PROPOSED SITES** ........ **25**
**15.0 TIMELINE** ........ ERROR! BOOKMARK NOT DEFINED.
**16.0 REFERENCES** ........ **26**
**APPENDIX I** ........ **30**
**APPENDIX III – CLINICAL TRIALS PROTOCOL REGISTRATION #** ........ **42**

## PROTOCOL SIGNATURE FORM

The signature of the Site Investigator below constitutes his/her approval of this protocol, and provides the necessary assurances that this study will be conducted according to all stipulations of the protocol, including all statements regarding confidentiality and in compliance with the current version of the protocol, International Conference on Harmonization Good Clinical Practice E6 (ICH-GCP), and the applicable national and local regulatory requirements.

____________________________________________________________________

Investigator's Name Signature of Investigator Date

# APPENDICES

**APPENDIX I** **Data and Safety Monitoring Committee**

**APPENDIX II** **Model Informed Consent**

**APPENDIX III** **Clinical Trials Protocol Registration**

## PROTOCOL ROSTER


**Protocol Co-Chair**
Jordan Lake, M.D
Clinical Instructor
UCLA CARE Center
David Geffen School of Medicine at UCLA
1399 S. Roxbury Drive
Los Angeles, CA 90035
P: 310-557-2273
F: 310-557-3450
jlake@mednet.ucla.edu

**Protocol Co-Chair**
Judith Currier, M.D.
Professor of Medicine
UCLA CARE Center
David Geffen School of Medicine at UCLA
9911 W. Pico Blvd., Suite 980
Los Angeles, CA 90035
P: 310-557-1891
F: 310-557-1899
jscurrier@mednet.ucla.edu

**UCLA**
Maricela Gonzalez
Operations Manager
1399 S. Roxbury Drive, Suite 100
Los Angeles, CA 90035
P: 310-557-3743
F: 310-557-3450
mmgonzalez@mednet.ucla.edu

Vanessa Cajahuaringa
Field Representative
1399 S. Roxbury Drive
Los Angeles, CA 90035
P: 310-557-3798
F: 310-557-3450
VCajahuaringa@mednet.ucla.edu

Carmen Malouf
Fund Manager
9911 W. Pico Blvd., Suite 980
Los Angeles, CA 90035
P: 310-557-3615

F: 310-557-1899
cmalouf@mednet.ucla.edu

Irma Franco-Gonzalez
Regulatory Administrator
9911 W. Pico Blvd., Suite 980
Los Angeles, CA 90035
P: 310-557-3675
F: 310-557-1899
ifranco@mednet.ucla.edu

Heather McCreath
Data Management Center
Division of Geriatrics, UCLA
10940 Wilshire, Suite 900
Los Angeles, CA 90024
P: 310-267-0848
F: 310-267-4257
hmccreath@mednet.ucla.edu

**CT Reading Center**

Andrea L. Desilets
Senior Research Coordinator
Body Composition Analysis Center
Tufts University Friedman School of Nutrition Science and Policy
150 Harrison Avenue, Jaharis Room #212
Boston, MA 02111
Email: Andrea.Desilets@tufts.edu
Phone: 617-636-3497
Fax: 617-636-3662

## 1.0 STUDY HYPOTHESIS AND OBJECTIVES:

### 1.1 Hypothesis

In HIV-positive subjects with central fat accumulation on ART, daily telmisartan therapy will be associated with a 10% reduction in visceral adipose tissue over 24 weeks.

### 1.2 Primary Objectives:

1.2.1 To assess the median change in visceral adipose tissue (VAT) volume (measured by abdominal CT scan) over 24 weeks in HIV-positive subjects with central fat accumulation initiating telmisartan therapy.

1.2.2 To describe the safety and tolerability of telmisartan in HIV-positive men and women over the 24 week duration of the study.

### 1.3 Secondary Objectives:

1.3.1 To assess changes in superficial adipose tissue (SAT) volume (by abdominal CT scan) over 24 weeks in HIV-positive subjects initiating telmisartan therapy.

1.3.2 To assess the change in VAT:SAT ration over 24 weeks in HIV-positive subjects initiating telmisartan therapy.

1.3.3 To assess the effects of telmisartan on serum lipid levels in HIV-positive subjects initiating telmisartan therapy.

1.3.4 To assess the effects of telmisartan on insulin sensitivity in HIV-positive subjects initiating telmisartan therapy.

1.3.5 To assess the effects of telmisartan on markers of inflammation in HIV-positive subjects initiating telmisartan therapy.

1.3.6 To compare Framingham risk scores at baseline and 24 weeks in HIV-positive subjects initiating telmisartan therapy.

1.3.7 To compare the prevalence of metabolic syndrome at baseline and 24 weeks in HIV-positive subjects initiating telmisartan therapy.

1.3.8 To measure changes in systolic and diastolic blood pressure in HIV-positive subjects initiating telmisartan therapy.

1.3.9 To assess changes in urinary protein excretion and serum creatinine in HIV-positive subjects initiating telmisartan therapy.

## 2.0 INTRODUCTION

### 2.1 Background and Rationale

In the context of HIV infection, the term lipodystrophy refers to a spectrum of changes in body fat redistribution and associated metabolic abnormalities, and is not precise[1-3]. Fat abnormalities can be categorized as either lipoatrophy (fat loss) or lipohypertrophy (fat gain), with each syndrome having unique risk factors, clinical features, and treatments. Fat redistribution in HIV-infected subjects has been reported to range from 2%-84%[2, 4-6], with the wide range reflecting a lack of consensus definition and diagnostic criteria.

Lipohypertrophy generally refers to fat accumulation in the abdomen, breasts, posterior neck (buffalo hump), and viscera. In the research setting, it is more specifically defined as a combination of patient-reported fat changes and an increased waist:hip ratio (>0.94 in men and >0.88 in women) or abdominal circumference (>95 cm in men and >94 cm in women)[7]. Cross-sectional imaging techniques such as computed tomography (CT) and magnetic resonance imaging (MRI) are the gold standard for measurement of body fat volume.

In observational and cross sectional studies, risk factors for lipohypertrophy have included protease inhibitor (PI) use, older age, white race, more advanced degree of immunosuppression, and female gender. However, recent prospective trials in treatment-naïve patients have called into question the role of PIs as a cause of lipohypertrophy[8]. After 48 weeks of tenofovir + lamivudine with either ritonavir-boosted tipranavir- or lopinavir, both peripheral and visceral fat decreased in both groups. After 48 weeks of either a tipranavir- or ritonavir/lopinavir (LPV/TRV)-based regimen combined with emtricitabine, visceral fat decreased in both groups. It is unknown whether a longer course of treatment with a PI might have contributed to the development of lipohypertrophy. In ACTG 5142, an efavirenz-based regimen induced the same level of trunk fat increase as that seen in LPV/RTV arm[9], again questioning the role of PIs in inducing the abdominal fat accumulation seen in HIV-infected subjects.

Lipohypertrophy is a common and distressing problem for people living with HIV infection. The decision to treat fat redistribution is frequently determined in the context of associated findings such as insulin resistance and hyperlipidemia, which can result from a combination of anti-retroviral therapy (ART) and visceral fat (VAT) accumulation. Currently, there is no consensus as to whether body fat changes should be treated in the absence of an associated metabolic syndrome. However, metabolic changes and aberrations of fat deposition may independently contribute to increased cardiovascular risk[9-12], making treatment of these states an important issue in the long-term management of HIV infection.

There are currently no FDA-approved treatments for central fat accumulation in HIV-infected patients, and efforts to combat HIV- and/or ART-related metabolic derangements and changes in fat distribution have met with mixed results. In part, this is due to an incomplete understanding of the mechanisms underlying these changes.

Metformin, which is used to improve insulin sensitivity in patients with diabetes and polycystic ovarian syndrome, has been shown in HIV infection to improve visceral fat accumulation but exacerbate peripheral lipoatrophy[13, 14], which may be an independent risk factor for CVD.

Both HIV and ART (specifically PIs and NRTIs) may modulate lipodystrophy via down-regulation of PPAR-γ[15,16]. Thiazolidinediones (TZDs), which also activate PPAR-γ, have not been shown to have an effect on VAT[17] in the setting of HIV infection, and have untoward side effects such as weight gain, fluid retention, and lipid abnormalities.

The effects of low-dose growth hormone on VAT are promising, but short-term negative effects on insulin sensitivity were observed, lipoatrophy worsened, its use requires patients to receive regular subcutaneous injections, and the effects appear to diminish quickly[18, 19]. Similarly, GH-Releasing Factor has been shown to decrease visceral fat and improve lipid profile, but has side effects similar to that of growth hormone, requires injection, and its effects also appear to diminish quickly[20].

Telmisartan is a renin-angiotensin system (RAS) antagonist (via angiotensin receptor type I inhibition) and partial PPAR-γ agonist approved for the treatment of essential hypertension. It has also been shown to decrease visceral fat volume, total cholesterol, and LDL levels, and improve fasting glucose levels, HDL, and markers of vascular inflammation in HIV-negative patients with the metabolic syndrome[21-23]. In subjects with mild to moderate renal insufficiency, telmisartan has been shown to decrease vascular intima-media thickness and improve renal function[24].

Telmisartan may exert it metabolic effects via multiple pathways.

**Insulin sensitivity**: The PPAR-γ and the RAS pathways are believed to affect insulin sensitivity via modulation of adipocyte function and differentiation[25], and both stimulate adiponectin secretion. Adiponectin decreases circulating lipid levels and improves insulin sensitivity via fatty acid catabolism and inhibition of gluconeogenesis in hepatocytes, and fatty acid oxidation and increased glucose uptake in skeletal muscle tissue[26]. Inhibition of adipocyte maturation via the RAS system promotes the deposition of fat into ectopic sites rather than mature adipocytes[25], improving insulin sensitivity. Additionally, telmisartan increases insulin-like growth factor I[27]. The improvement in insulin sensitivity seen with telmisartan usage may also be, in part, a secondary effect of fat remodeling (see below), as insulin resistance is known to be associated with visceral adiposity[28, 29]. Lastly, in animal models, an additional adipose tissue-independent mechanism for insulin sensitivity has been suggested for telmisartan[30].

**Vascular Inflammation**: Telmisartan may decrease vascular inflammation by: improving glucose-induced oxidative stress, PPAR-γ-independent TNF-alpha inhibition, suppression of IL-6, and RAS-mediated nitric oxide synthase modulation[31-34].

**Weight loss**: In both humans and animal models, weight loss with telmisartan usage has been observed, an effect postulated to occur via an anorexigenic hypothalamic effect[35-37]. Decreases in BMI have been shown to correspond with improved serum leptin levels, signifying the loss of adipose tissue[38].

**Visceral Fat:** Telmisartan may decrease VAT accumulation via multiple mechanisms. First, it has been hypothesized that telmisartan has broad metabolic effects on adipose tissue, including decreased adipocyte size, and may improve caloric metabolism[35]. Second, telmisartan directly stimulates adipocyte maturation, including subcutaneous adipocytes, preparing them for lipid uptake[39]. Third, PPAR-γ agonism may initiate redistribution of fat from visceral to subcutaneous depots, which is a purported mechanism of the TZD class of agents[40]. Lipid uptake into subcutaneous depots (and movement out of visceral depots) leads to increased energy requirements/metabolism of visceral adipocytes, the end result of which is decreased VAT accumulation[41]. Thus, telmisartan directly acts on adipose tissue and decreases visceral fat accumulation via mechanisms independent of its primary insulin sensitizing effects.

Telmisartan has been studied at standard doses in both hypertensive and normotensive patients, and has been shown to have an excellent safety profile in both populations[42]. It has also been shown to bind PPAR-γ differently than the thiazolidinediones[43], which may explain its improved tolerability.

Telmisartan has not been studied in HIV-positive patients to date, but is an excellent candidate for the treatment of HIV- and ART-related metabolic dysregulation. Its potential effects on visceral fat, lipid levels, and cardiovascular risk are of particular interest, as current therapeutic options remain limited and of unclear benefit. If improvement in these parameters could be demonstrated in HIV-positive patients, telmisartan would be the only agent capable of treating multiple metabolic derangements (hypertension, hyperlipidemia, insulin resistance) in HIV-infected persons, and would decrease: pill burden, risk of drug interactions, adverse events.

The goal of this study is to evaluate the safety and tolerability of telmisartan in virologically-suppressed HIV-positive subjects with central fat gain during effective ART.

## 3.0 STUDY DESIGN

Single arm, open-label, 24-week study of the addition of telmisartan 40mg po daily to continued ART in subjects with central fat accumulation and suppressed HIV-1 RNA on a stable anti-retroviral regimen.

## 4.0 SELECTION AND ENROLLMENT OF SUBJECTS

### 4.1 Inclusion Criteria

4.1.1 HIV positive men and women age 18 and older.

4.1.2 HIV-1 infection, as documented by any licensed ELISA test kit and confirmed by Western blot at any time prior to study entry or plasma HIV-1 RNA > 2000 on two occasions**.**

4.1.3 Documented central fat accumulation (defined for women/men as: waist circumference > 94/95 cm or a waist-to-hip ratio > 0.88/0.94).

4.1.4 HIV-1 RNA documented to be less than 50 copies/mL at screening and undetectable by assay of choice (<50 or <400) for at least 12 weeks prior to entry**.**

4.1.5 Current antiretroviral therapy with a suppressive, highly active anti-retroviral regimen. Subjects must not have changed ART in the 12 weeks prior to entry, and must not be planning to change ART for the 24 week study duration.

4.1.6 Systolic blood pressure >115mmHg. Telmisartan has been demonstrated to be safe for use in normotensive subjects.

4.1.7 Ability and willingness of subject to provide informed consent.

### 4.2 Exclusion Criteria

4.2.1 Pregnancy (current or within the past 6 months) or nursing.

Note: For females of reproductive potential [defined as women who have had menses in the 12 months prior to entry, or who have not undergone surgical sterilization (hysterectomy, bilateral oophorectomy, and/or tubal ligation)], will need a negative serum or urine pregnancy test in the 48 hours prior to entry. If participating in sexual activity that could lead to pregnancy, study participants must use contraception while on study drug and for 4 weeks after going off study drug.

4.2.2 Uncontrolled hypertension. If subjects are currently on a stable anti-hypertensive regimen, they must be willing to add telmisartan to their current regimen and provide documentation from their cardiologist or prescribing physician that the addition of telmisartan is acceptable/not contraindicated. To minimize the potential for aggravation of renal function, if subjects require titration of their antihypertensive regimen while on study, it is asked that ACE inhibitors not be titrated unless other options are not available.

4.2.3 Prohibited concomitant medications: Thiazolidinediones, other members of the angiotensin receptor-blocking class (losartan, irbesartan, olmesartan, valsartan, candesartan), nelfinavir, etravirine. Subjects taking nelfinavir or etravirine will be excluded due to the possibility of increased drug levels via inhibition of cytochrome P-450 2C19.

Note: Subjects requiring amifostine or rituximab must be aware of the increased risk of orthostatic hypotension with the addition of telmisartan. Any subject requiring lithium therapy while on study must have lithium levels monitored closely by their outside physician. All subjects on the above listed medications should provide documentation that this physician is aware of the study protocol.

4.2.4 Subjects with untreated hyperlipidemia must be willing to abstain from initiating therapy for the 24 week duration of the study. Subjects on a stable (>12 weeks) lipid-lowering regimen must be willing to remain on their current dose for the 24 week study duration.

4.2.5 Subjects undergoing treatment for diabetes with oral hypoglycemic agents must be willing to remain on their current dose of insulin-sensitizing agents (metformin/biguanides) for the duration of the study. Titration of other diabetes medications will be permitted.

4.2.6 Screening laboratory values as follows:

- -ANC < 750 cells/mm3
- -Hemoglobin <10 gm/dL
- -Creatinine clearance<30 ml/min (estimated by Cockcroft-Gault equation using ideal body weight)
- -AST or ALT > 3 times ULN

4.2.7 Known, untreated renal artery stenosis.

4.2.8 Unstable coronary artery disease/angina or decompensated congestive heart failure.

4.2.10 Any history of intolerance to any member of the angiotensin receptor blocker class of agents.

4.2.11 Need for ongoing potassium supplementation.

## 5.0 STUDY TREATMENT

### 5.1 Regimens, Administration, and Duration

Study treatment is open label telmisartan.

All subjects will receive telmisartan 40 mg po daily for the 24 week study duration.

For the purposes of managing hypotension or maintaining stable renal function, if a subject's sbp becomes <100mmHg or serum creatinine significantly increases (in the eyes of the investigator) while on telmisartan therapy, a dose reduction to 20mg po daily will be allowed.

### 5.2 Study Product Formulation and Preparation

Telmisartan 40mg tablets must be dispensed in the original bottle with the desiccant provided. Store at room temperature (25°C, 77°F) and protect from moisture.

### 5.3 Pharmacy: Product Acquisition, Distribution, and Accountability

<u>Study Product Acquisition/Distribution</u>
Telmisartan will be purchased for the study following IRB approval. The use of telmisartan in this setting qualifies for an IND waiver, which will be obtained in a timely fashion.

Telmisartan will be provided at no cost to the subject. However, subjects will remain responsible for providing all other needed medications (including but not limited to ART).

### 5.4 Concomitant Medications

<u>Prohibited Medications</u>
The prohibited medications with telmisartan are listed in Section 4.2.3.

## 6.0 CLINICAL EVALUATIONS

### 6.1 Schedule of Events

| | Screen | Baseline* | Week 1 | Week 2 | Week 6 | Week 12* | Week 18 | Week 24* |
|---|---|---|---|---|---|---|---|---|
| Informed Consent, Patient's Bill of Rights, HIPPA, medical information release | X | | | | | | | |
| Targeted physical exam with weight, blood pressure | | X | X | X | X | X | X | X |
| Complete physical exam and height | X | | | | | | | |
| CT- L4/5 | | X | | | | | | X |
| Mid-waist, hip and neck circumferences and waist/hip ratio | X | X | | | | | | X |
| HIV-1 RNA | X | X | | | | | | X |
| Safety labs** | X | X | | | X | X | X | X |
| CD4 cell counts | | X | | | | | | X |
| Fasting lipids, hsCRP, banked serum and plasma | | X | | | | X | | X |
| Urine samples for markers of oxidant stress and endothelial activation, protein | | X | | | | X | | X |

* Entry, week 12, and week 24 laboratory evaluations are required to be fasting (at least 8 hours).

**Safety labs include: CBC with differential, chemistry panel [including liver enzymes (ALT, AST, Alk Phos) and total bilirubin, serum creatinine], urine or serum pregnancy test. All safety labs, HIV-1 RNA, and CD4 cell counts will be done at local certified laboratories.

### 6.2 Timing of Study Evaluations

**Visit I Screening**

After obtaining informed consent, study participants will undergo a series of screening evaluations to determine eligibility. The waist and hip measurements will be performed first to determine if the subject meets criteria (defined for women/men as: waist circumference > 94/95 cm or a waist-to-hip ratio > 0.88/0.94). All sites will be trained in the procedures for obtaining waist and hip measurements, and these will be collected in triplicate.

If eligible, the subject will undergo a single 5 mm slice CT scan at the level of L4-L5, and the image will be sent to the central reading center in electronic format. The CT scan will not be scheduled until eligibility is confirmed by exam and laboratory parameters.

**Visit 2 Baseline (Week 0, within 30 days of screening)**

Once eligibility has been confirmed, the subject will be scheduled for baseline evaluation, which includes safety lab evaluation and the collection of stored serum, plasma, and urine samples for future analyses. Telmisartan 40 mg po daily will be distributed at this evaluation, and the subject will be counseled (again) regarding adherence and potential medication side effects.

For all subsequent visits, evaluations should be performed +/- 14 days from the expected date. Medication adherence/compliance and method of birth control (if applicable) will be assessed and documented at every visit.

**Visits 3 and 4 (Weeks 1 and 2)**

These visits will assess blood pressure and any subject-reported adverse events 7 and 14 days, respectively, following initiation of telmisartan. No laboratory evaluations will be performed unless prompted by adverse events.

**Visit 5 (Week 6)**

A targeted physical exam (including blood pressure measurement) and safety laboratory evaluations will be performed.

**Visit 6 (Week 12)**

This visit will include a targeted physical exam, safety laboratory evaluation, and the collection of stored serum, plasma, and urine samples for future analyses.

**Visit 7 (Week 18)**

A targeted physical exam and safety laboratory evaluations will be performed.

**Visit 8 (Week 24)**

This visit will be the final study visit. A targeted physical exam, safety laboratory evaluation, and the collection of banked serum, plasma, and urine samples will be performed. A single 5 mm slice CT scan at the level of L4-L5 will be scheduled within 14 days of this visit, and the image will be sent to the central reading center in electronic format. After this visit, telmisartan will no linger be provided by the study. However, should a subject wish to continue to take telmisartan, he or she may do so by prescription from their primary care provider.

### 6.3 Pregnancy

Subjects who become pregnant after study entry must discontinue study treatment immediately. Subjects will continue to be followed on study but off study treatment as per the Schedule of Events in Section 6.1, except for CT scans. Pregnancies that occur on study should be reported to The Antiretroviral Pregnancy Registry. Reporting forms can be found in the Manual of Operations (MOP). More information is available at www.apregistry.com. Phone: 800-259-4263;
Fax: 800-800-1052.

### 6.4 Discontinuation Evaluations

Evaluations for Registered Subjects Who Do Not Start Study Treatment
Subjects who do not start study treatment will be taken off study without further evaluation. The subject will be replaced. All case report forms (CRFs) must be completed and keyed for the period up to and including week 0.

Premature Study Treatment/Background ART Discontinuation Evaluations
Subjects who discontinue telmisartan due to adverse events or by personal choice will be followed and asked to complete the Week 12 and Week 24 evaluations (as listed in the Schedule of Events (section 6.1)) prior to being taken off study.

Subjects who discontinue their background ART entry regimen for ≥14 consecutive days due to toxicity or adherence will be asked to complete the Week 12 and Week 24 evaluations prior to being taken off study.

Subjects experiencing drops in systolic blood pressure below 100mmHg (without sequelae) or asymptomatic increases in serum creatinine, AST, ALT, or alkaline phosphatase (to <3x ULN for liver enzymes) may temporarily discontinue telmisartan. When systolic blood pressure returns to >100mmHg and/or if laboratory abnormalities resolve within 14 days, telmisartan may be restarted at the discretion of the study physician and subject at a dose of 20 mg po daily. The subject should be monitored closely for tolerability. If the subject does not experience a recurrence of the adverse event, he/she may continue on telmisartan for the duration of the study.

### 6.5 Clinical Assessments

Documentation of HIV
HIV-1 infection, as documented by any licensed ELISA test kit and confirmed by Western blot at any time prior to study entry. HIV-1 culture, HIV-1 antigen, plasma HIV-1 RNA, or a second antibody test by a method other than ELISA are acceptable alternative confirmatory tests. This does not need to be recorded on a CRF, but a copy should be placed in the subjects study chart for documentation.

Documentation of Virologic Suppression

HIV-1 viral load at screening must be less than 50 copies/mL. Documentation should be provided in the study chart that the subject has had an undetectable viral load for at least 12 weeks prior to entry, but does not need to be recorded on CRFs. Documentation of viral loads other than the screening value shall be defined using the assay in use at the time (<50 copies/mL or <400 copies/mL).

Medical History
The medical history must include all AIDS-related diagnoses and any history of coronary artery disease, congestive heart failure, renal disease, hypertension, diabetes, or stroke. ACTG diagnostic criteria will be provided in the Manual of Operations to aid in documentation. Any allergies to any medications and their formulations must be documented. Surgeries that are planned prior to the time of the subject's informed consent will also be documented. Current and previous smoking history will also be collected.

Medication History
A medication history must be recorded in the source documents and the CRF. The medication history will include the following:

- Complete HIV treatment history, including start and stop dates of any current or past antiretroviral medication (estimated if the exact dates cannot be obtained), immune-based therapy, or HIV-related vaccines, including blinded study medications.
- Complete treatment history of any prescription medications taken for the treatment or prophylaxis of opportunistic infections, including actual or estimated start and stop dates.
- All prescription medications (in addition to those noted above) taken in the 30 days prior to study entry, including actual or estimated start and stop dates. Only dosages for ART will be required.
- Nonprescription medications taken in the 30 days prior to study entry. Include actual or estimated start and stop dates.
- Alternative therapies and dietary supplements taken in the 30 days prior to study entry. Include actual or estimated start and stop dates.

Complete Physical Exam
A complete physical examination is required at screening and includes: examination of the skin, head, mouth, and neck; auscultation of the chest; cardiac exam; abdominal exam; examination of the lower extremities for edema. The exam also includes measurement of mid-waist and hip circumferences, a waist/hip ratio calculation, and documentation of signs and symptoms, diagnoses, vital signs (temperature, pulse, respiration rate, and blood pressure), height, and weight. In addition, the exam includes an assessment (and subject reported changes) of neck fat, breast enlargement, lipomas, and peripheral lipoatrophy.

Targeted Physical Exam
A targeted physical examination at visits for Weeks 0-24 is to be driven by any previously-identified or new signs or symptoms that the subject has experienced since the last visit. Weight, vital signs (temperature, pulse, respiration rate, and blood pressure), and diagnoses will also be collected at all visits.

Height
Height in stocking feet will be recorded on the CRF at the screening visit only.

Signs and Symptoms
At entry, record all signs and symptoms occurring in the 30 days prior to entry. After entry, record all signs and symptoms ≥ Grade 2. This study will use the DAIDS toxicity grading system (see Manual of Operations). Any signs or symptoms that led to a change in the study drug (telmisartan), regardless of grade, must be recorded.

Diagnoses
Record all diagnoses identified by the ACTG Criteria for Clinical Events and Other Diseases (see Manual of Operations). All confirmed and probable diagnoses made since the last visit will be recorded.

Concomitant Medications
All concomitant medications taken since the last visit will be recorded as a running list in the source documents. Only prescription medications and method of birth control (where applicable) will be recorded on the CRFs.

Antiretroviral Medications
All modifications to antiretroviral medications including doses and subject- or physician-initiated interruptions, modifications, or permanent discontinuation will be recorded on the CRFs.

Study Treatment Modifications
Modifications to telmisartan dosing, schedule, interruptions, or permanent discontinuation will be recorded on the CRFs. If a subject misses more than 4 consecutive doses, the study treatment is considered modified and this must be reported appropriately on the CRF.

CT Scan
Single-slice CT abdomen at the level of L4/L5, with measurement of visceral and subcutaneous fat, will be read centrally by Tufts University. Please see Manual of Operations for instructions.

### 6.6. Laboratory Evaluations
Fasting will be required for study entry, Week 12, and Week 24. Fasting will be defined as nothing by mouth except water and medications for at least 8 hours prior to the lab draw. At screening and entry, all laboratory values must be recorded on the CRFs regardless of grade. For post-entry assessments, record all laboratory values ≥ Grade 2. Any laboratory value that led to a change in study treatment (telmisartan), regardless of grade, must be recorded. Fasting lab studies include: total cholesterol, LDL cholesterol, triglycerides, HDL cholesterol, glucose, and insulin.

**Sites must refer to the Division of AIDS Table for Grading the Severity of Adult and Pediatric Adverse Events** (see Manual of Operations), **Version 1.0, December 2004, to establish grading.** The table is available on the telmisartan study website at http://www.uclacarecenter.org/MATHtrial.org.

Pregnancy Test
For women with reproductive potential: Serum or urine β-HCG (urine test must have a sensitivity of 25-50 mIU/mL) must be performed at each safety evaluation time point, and/or whenever pregnancy is suspected.

Fasting lipids, Glucose, Insulin, hsCRP
Total cholesterol, HDL cholesterol, LDL cholesterol (direct), triglycerides, hsCRP, and glucose will be performed at entry and weeks 12 and 24 in real time at the local laboratory. Insulin will be batched and analyzed at the end of the study.

Plasma HIV-1 RNA
HIV-1 RNA must be performed by a laboratory that possesses a CLIA certification or equivalent. Eligibility will be determined based on a screening value of <50 copies/mL.

CD4+ Count
Obtain absolute CD4+/CD8+ counts and percentages. All laboratories must possess a CLIA certification or equivalent.

Safety Labs
CBC with differential, chemistry panel including liver enzymes (ALT, AST, Alk Phos), total bilirubin, serum creatinine, and urine or serum pregnancy test. All safety labs, HIV-1 RNA, and CD4 cell counts will be done at local certified laboratories.

Fasting Stored Serum, Plasma and Urine
If subjects come to the appointment and are not fasting, they will need to come back in fasting state for these evaluations within a 7-day window.

Serum (3 mL), plasma (3 mL), and clean-catch urine (minimum of two and maximum of six 3 mL aliquots. Please see Manual of Operations for further details) should be collected for storage as indicated in the schedule of events (entry, Week 12, Week 24). An additional set of banked samples (identical in volume to above) should be collected in the event of study discontinuation. Frozen specimens will be collected, labeled with patients' PID and date of draw, and stored in a –70 degrees freezer (urine specimens may be refrigerated up to 24 hours prior to freezing if needed) until analyses can be performed. The stored samples will be used for future measurements of markers of inflammation and glucose/lipid regulation such as CRP, TNF-α, soluble TNF receptors I and II, IL-6, adiponectin, leptin, insulin, HOMA-IR, myeloperoxidase (MPO), prostaglandin metabolites.

## 7.0 TOXICITY MANAGEMENT
Criteria for subject management and dose interruptions, modifications, or discontinuation will pertain only to toxicities attributable to telmisartan. Toxicities due to drugs in the background regimen should be managed according to standard clinical practice, with the goal of maintaining continuous therapy, if possible.

### 7.1. Grade 1 or 2 Toxicity
Subjects who develop a Grade 1 or 2 AE or toxicity may continue telmisartan.

### 7.2 Grade 3 Toxicity
Subjects who develop a Grade 3 AE or toxicity should have telmisartan withheld unless the investigator has compelling evidence that the AE was not caused by telmisartan. The study chairs

(Drs. Currier or Lake) should be notified of any Grade 3 or greater toxicity. The subject should be re-evaluated closely until the AE returns to Grade ≤ 2, at which time telmisartan may be reintroduced at the discretion of the site investigator or according to standard practice.

If the same Grade 3 AE recurs within 4 weeks and it is thought to be related possibly, probably, or definitely to telmisartan, then telmisartan must be permanently discontinued. If the same Grade 3 AE recurs after 4 weeks but is not thought to be related to telmisartan, the management scheme outlined above may be repeated.

Subjects experiencing Grade 3 or greater AEs (requiring permanent discontinuation of telmisartan) should be followed closely for resolution of the AE to Grade ≤2, and the study chairs must be consulted.

Subjects with Grade 3 asymptomatic laboratory abnormalities in cholesterol or triglycerides may continue telmisartan. Grade 3 asymptomatic elevations of bilirubin in subjects on atazanavir therapy will also be permitted.

### 7.3 Grade 4 Toxicity

Subjects who develop a Grade 4 symptomatic AE or toxicity will have telmisartan discontinued. If the site investigator has compelling evidence that the AE has not been caused by telmisartan, dosing may resume when the AE has resolved and after consulting with the study chairs. Subjects experiencing Grade 4 AEs requiring permanent discontinuation of telmisartan should be followed closely until resolution of the AE to Grade ≤2, and the study team must be consulted.

Subjects with Grade 4 asymptomatic laboratory abnormalities in cholesterol or triglycerides may continue telmisartan.

## 8.0 CRITERIA FOR DISCONTINUATION

### 8.1 Permanent Study Drug Discontinuation

Study drug discontinuation can occur for the following reasons:

- Drug-related toxicity requiring permanent discontinuation (see Sections 7.2, 7.3).
- Requirement for prohibited concomitant drugs (see Section 4.2.3).
- Request by subject to terminate treatment.
- Confirmation of virologic failure (two measurements of HIV RNA > 50 copies/mL at least 2 weeks apart).
- Clinical reasons believed life-threatening by the physician, even if not addressed in the toxicity section of the protocol.
- Subject repeatedly noncompliant with telmisartan as prescribed.
- Subject discontinues telmisartan for ≥14 consecutive days.
- Failure by the subject to attend ≥3 consecutive clinic visits.
- Pregnancy or breast-feeding.

### 8.2 Premature Study Discontinuation

- Request by the subject to withdraw.

- Request of the primary care provider if he or she thinks the study is no longer in the best interest of the subject.
- Subject judged by the investigator to be at significant risk of causing harm to self.
- At the discretion of the IRB, Food and Drug Administration (FDA), Office for Human Research Protections (OHRP), investigator, or the pharmaceutical sponsor.

**8.3 Early Discontinuation**
Any subject who develops a Grade 3 or 4 symptom or laboratory abnormality (other than cholesterol, and triglycerides), UNLESS THE INVESTIGATOR HAS A COMPELLING REASON TO THINK IT IS NOT RELATED TO TELMISARTAN) will be evaluated and will have study treatment withheld until the toxicity resolves ($\leq$ grade 2). If the abnormality is thought to be related to study treatment, the subject will not be re-challenged without discussion with protocol chairs (see Sections 7.2, 7.3). Subjects requiring premature study discontinuation will undergo a final safety evaluation and CT scan. Serum and plasma will also be banked at the time of study discontinuation (in lieu of the Week 12 or Week 24 sample). All subjects will remain in follow-up for resolution of Grade 3 or 4 toxicities.

## 9.0 SAMPLE SIZE AND ENDPOINTS

**9.1 Sample Size**
In a recently reported study of growth hormone releasing factor in HIV lipodystrophy[7], the FDA determined an 8% change in visceral abdominal fat between the active and placebo arms would define a clinically meaningful difference in VAT volume, assuming a standard deviation of 18.5%. However, we will aim for 10% difference in visceral fat to make it more clinically significant.

Using the parameters recommended by the FDA, we estimate that a sample size of 27 subjects will provide 80% power to detect a 10% reduction in VAT over 24 weeks in subjects treated with telmisartan (two-sided alpha level=0.05). We have increased the sample size to 35 subjects to improve our power to look at secondary endpoints, and to account for potential loss of follow-up. The total sample size is 35 subjects.

**9.2 Randomization and Stratification**
Subjects will not require randomization as this pilot study is a single arm, open label trial. Stratification will occur by gender.

**9.3 Primary Endpoint Assessments**
VAT volume ($cm^2$) will be calculated from a single 5 mm slice CT scan obtained at the level of the L4-L5 inter-vertebral disc space. CT scans will be obtained prior to telmisartan initiation and at study termination (Weeks 0 and 24). All CT scans will be standardized and communicated via electronic medium (CD, DVD, or optic disk) to the central reading center at Tufts University. The central reader will be blinded to patient characteristics.

**9.4 Secondary Endpoint Assessments** (Change in below calculated between Weeks 0 and 24)

- Abdominal SAT and VAT:SAT ratio, as calculated from single slice CT scan (as above)
- Total cholesterol, HDL cholesterol, triglycerides, LDL cholesterol (direct)
- Markers of inflammation

- Insulin, glucose, HOMA-IR
- Framingham Risk Score calculation
- Prevalence of metabolic syndrome
- Systolic and diastolic blood pressure measurements
- Urinary protein excretion and serum creatinine measurement

### 9.5 Study Monitoring

This is an open label study. Accrual and a summary of all Grade ≥2 signs and symptoms and all Grade ≥3 laboratory abnormalities will be prepared by the central data management site at UCLA, and reviewed by the team monthly. Baseline characteristics, early treatment discontinuations, and study discontinuations will be reviewed by the protocol chairs. The data management center at UCLA will also prepare a quarterly report of all AEs for review.

An independent Data Safety Monitoring Board (DSMB) comprised of three HIV investigators not involved in the study will review accrual, AE summaries, off-treatment, and off-study rates quarterly. A DSMB review may also be convened if a reason is identified by the study chairs, the data management center at UCLA, or the pharmaceutical sponsor.

### 9.6 Analyses

All statistical tests will be two-sided with nominal level of 0.05. Analysis will be exploratory without adjusting for multiple testing.

9.6.1 Primary Analysis

Comparison of VAT volume change between Weeks 0 and 24 will be assessed using the paired t test. The primary analysis will be as-treated, excluding subjects who do not remain on the study regimen and/or subjects who do not have an observed primary endpoint. Subjects who discontinue telmisartan for ≥ 14 consecutive days will not contribute to the primary outcome after the date they first discontinued medication. An intent-to-treat analysis will be performed as a supplement.

9.6.2 Occurrence of Study-Related AEs

The number of subjects who experience treatment-related AEs between Weeks 0 and 24 will be reported.

9.6.3 Proportion of Subjects who Discontinue Study Treatment

The proportion of subjects who discontinue study treatment prior to Weeks 12 and 24 will be summarized. Reasons for discontinuing study drug (telmisartan) will be listed.

## 10.0 DATA COLLECTION, MONITORING, AND ADVERSE EVENT REPORTING

### 10.1 Records to Be Kept

Data will be collected onto Case Report Forms (CRFs) provided by the Data Management Center at UCLA, and will be labeled only with a subject's study ID number. CRFs will be stored in a locked cabinet available only to study personnel for a period of time determined by pertinent policies and regulations, and for at least two years after study discontinuation.

### 10.2 Role of Data Management

Instructions regarding the recording of study data on CRFs and entry into the online data management system will be provided by the data management center. All data will be entered into the online system within 14 days.

### 10.3 Clinical Site Monitoring and Record Availability

Heather McCreath from the UCLA Data Management Center will perform an on-site review of individual subject records, including: consent forms, CRFs, supporting data, laboratory specimen records, and medical records (physicians' progress notes, nurses' notes, individuals' hospital charts). Such monitoring will ensure protection of study subjects, compliance with the protocol, and accuracy and completeness of records. The monitor also will inspect regulatory files to ensure requirements are being met. Finally, pharmacy drug storage and dispensing records will be reviewed to ensure appropriate pharmaceutical product storage and management.

### 10.4 Serious Adverse Event Reporting

The serious adverse event (SAE) reporting requirements and definitions for this investigator-initiated study, and the methods for reporting of SAEs to the Regulatory Center at the University of California at Los Angeles (UCLA) are defined according to UCLA policies and regulations. Standard post-marketing reporting of SAEs will be performed to the maker of the drug, Boehringer Ingelheim. Guidelines for SAE reporting are included in the MOP and available on the UCLA MATH study website: http://www.uclacarecenter.org/MATHtrial.html.

The study agent for SAE reporting is telmisartan.

SAEs must be documented on the Serious Adverse Event Reporting form (available in the MOP) and on the MATH study website: http://www.uclacarecenter.org/MATHtrial.html.

The study chairs (Drs. Currier or Lake) must be notified of any Grade 3 or greater toxicity that does not have an etiology clearly documented as not related to telmisartan.

All Grade 4 events, death, persistent or significant disability, incapacity, hospitalization, prolongation of hospitalization, or event that the site investigator deems medically significant and related possibly, probably, or definitely to telmisartan must be reported by completing and faxing the SAE Reporting form to (310) 557-1899.

Serious Adverse Events must be reported during the protocol-defined SAE Reporting period, which is from enrollment until four weeks following the subject's last dose of study drug.

After the end of the protocol-defined SAE Reporting Period (stated above), sites must report serious, unexpected, clinically-suspected adverse drug reactions if the study site staff becomes aware of the event on a passive basis, i.e., from publicly available information.

The DSMB will be notified of any serious AEs in real time, and will also receive quarterly SAE reports for review.

## 11.0 HUMAN SUBJECTS

### 11.1 Institutional Review Board (IRB) Review and Informed Consent

This protocol, the informed consent document (Appendix II), and any subsequent modifications will be reviewed and approved by the IRB. The Patient's Bill of Rights will be reviewed with the subject prior to initiation of the informed consent process. A signed consent form will be obtained from the subject prior to screening or other study procedures. Re-consent will be obtained prior to study continuation should protocol modifications be required leading to the development of an amended informed consent document. The consent form will describe the purpose of the study, the procedures to be followed, and the risks and benefits of participation. A copy of the signed consent form will be given to the subject, and this fact will be documented in the subject's record.

### 11.2 Subject Confidentiality

All laboratory specimens, evaluation forms, reports, and other records that leave the site will be identified by coded number only to maintain subject confidentiality. All records will be kept locked. All computer entry and networking programs will be done with coded numbers only. Clinical information will not be released without written permission of the subject, except as necessary for monitoring by IRB, DSMB, FDA, OHRP, or the pharmaceutical sponsor or their designee. All subjects will sign and receive a copy of HIPPA regulations prior to study commencement. The original will be placed in the study chart, and a copy will be given to the subject.

### 11.3 Study Discontinuation

The study may be discontinued at any time by the IRB, pharmaceutical supporter, FDA, OHRP, or other government agencies when necessary to ensure the protection of research subjects.

## 12.0 PUBLICATION OF RESEARCH FINDINGS

Drs. Currier and Lake will be responsible for the dissemination of study results in the form of presentations, abstracts, or manuscripts

## 13.0 Biohazard Containment

As the transmission of HIV and other blood-borne pathogens can occur through contact with contaminated needles, blood, and blood products, appropriate blood and secretion precautions will be employed by all personnel in the drawing of blood, shipping, and handling of all study specimens (as currently recommended by the Centers for Disease Control and Prevention and the National Institutes of Health).

**All dangerous goods and materials, including diagnostic specimens and infectious substances, must be transported according to the International Air Transport Association (IATA) Dangerous Goods Regulations.**

## 14.0 PROPOSED SITE

The UCLA CARE Center has experience conducting metabolic studies, HIV expertise, and access to an appropriate subject pool. It will serve as the single site for the study.

## 15.0 REFERENCES


1. Bacchetti P, Gripshover B, Grunfeld C, et al. *Fat distribution in men with HIV infection*. J Acquir Immune Defic Syndr. Oct 1 2005;**40**(2):121-131.

2. Carr A. *HIV lipodystrophy: risk factors, pathogenesis, diagnosis and management*. AIDS. Apr 2003;**17** Suppl 1:S141-148.

3. Miller J, Carr A, Emery S, et al. *HIV lipodystrophy: prevalence, severity and correlates of risk in Australia*. HIV Med. Jul 2003;**4**(3):293-301.

4. Gervasoni C, Ridolfo AL, Trifiro G, et al. *Redistribution of body fat in HIV-infected women undergoing combined antiretroviral therapy*. AIDS. Mar 11 1999;**13**(4):465-471.

5. Saves M, Raffi F, Capeau J, et al. *Factors related to lipodystrophy and metabolic alterations in patients with human immunodeficiency virus infection receiving highly active antiretroviral therapy*. Clin Infect Dis. May 15 2002;**34**(10):1396-1405.

6. Wanke C, Gerrior J, Kantaros J, Coakley E, Albrecht M. *Recombinant human growth hormone improves the fat redistribution syndrome (lipodystrophy) in patients with HIV.* AIDS. Oct 22 1999;**13**(15):2099-2103.

7. Falutz J, Allas S, Blot K, et al. *Metabolic effects of a growth hormone releasing factor in patients with HIV.* N Engl J Med. Dec 6 2007;**357**(23):2359-2370.

8. Carr et al. *Effects of boosted tipranavir and lopinavir on body composition, insulin sensitivity, and adipocytokines in anti-retroviral naïve adults*. AIDS. 2008 Nov 12;22(17):2313-21.

9. Haubrich RH, Riddler S, DiRienzo G, Komarow L, Powderly W, Garren K, George T, Rooney J, Mellors J, Havlir D, and the AIDS Clinical Trials Group 5142 Study Team. *Metabolic outcomes in a randomized trial of nucleoside, non-nucleoside-and protease inhibitor-sparing regimens for initial HIV treatment*. AIDS. 2009 Jun 1;**23**(9):1109-18.

10. Mulligan K, Grunfeld C, Tai VW, et al. Hyperlipidemia and insulin resistance are induced by protease inhibitors independent of body fat changes in HIV infection. *J Acquir Immune Defic Syndr*. 2000; 23:35-43.

11. Wohl et al. The association of regional adipose tissue with lipid and lipoprotein levels in HIV-infected men. *J Acquir Immune Defic Syndr*. 2008; 48:44-52.

12. Currier et al. Regional adipose tissue and lipid and lipoprotein levels in HIV-infected women. *J Acquir Immune Defic Syndr*. 2008; 48:35-43.

13. Hadigan, C., et al., *Metformin in the treatment of HIV lipodystrophy syndrome: A randomized controlled trial.* JAMA, 2000. **284**(4): p. 472-7.

14. Kohli, R., et al., *A randomized placebo-controlled trial of metformin for the treatment of HIV lipodystrophy.* HIV Med, 2007. **8**(7): p. 420-6.

15. Caron, M., et al., *Antiretroviral-Related Adipocyte Dysfunction and Lipodystrophy in HIV-Infected Patients: Alteration of the PPARgamma-Dependent Pathways.* PPAR Res, 2009. **2009**: p. 507141.

16. Lemoine, M., J. Capeau, and L. Serfaty, *PPAR and Liver Injury in HIV-Infected Patients.* PPAR Res, 2009. **2009**: p. 906167.

17. Gelato, M.C., et al., *Improved insulin sensitivity and body fat distribution in HIV-infected patients treated with rosiglitazone: a pilot study.* J Acquir Immune Defic Syndr, 2002. **31**(2): p. 163-70.

18. Lo, J., et al., *Low-dose physiological growth hormone in patients with HIV and abdominal fat accumulation: a randomized controlled trial.* JAMA, 2008. **300**(5): p. 509-19.

19. Macallan, D.C., et al., *Treatment of altered body composition in HIV-associated lipodystrophy: comparison of rosiglitazone, pravastatin, and recombinant human growth hormone.* HIV Clin Trials, 2008. **9**(4): p. 254-68.

20. Falutz J, et al. *Long-term safety and efficacy of tesamorelin, a growth hormone releasing factor analogue, in HIV patients with abdominal fat accumulation*. AIDS 2008, **22(**14**)**:1719-1728.

21. Shimabukuro, M., H. Tanaka, and T. Shimabukuro, *Effects of telmisartan on fat distribution in individuals with the metabolic syndrome.* J Hypertens, 2007. **25**(4): p. 841-8.

22. Chujo, D., et al., *Telmisartan treatment decreases visceral fat accumulation and improves serum levels of adiponectin and vascular inflammation markers in Japanese hypertensive patients.* Hypertens Res, 2007. **30**(12): p. 1205-10.

23. Derosa, G., et al., *Metabolic effects of telmisartan and irbesartan in type 2 diabetic patients with metabolic syndrome treated with rosiglitazone.* J Clin Pharm Ther, 2007. **32**(3): p. 261-8.

24. Nakamura, T., et al., *Comparison of renal and vascular protective effects between telmisartan and amlodipine in hypertensive patients with chronic kidney disease with mild renal insufficiency.* Hypertens Res, 2008. **31**(5): p. 841-50.

25. Sharma, A.M., *The obese patient with diabetes mellitus: from research targets to treatment options.* Am J Med, 2006. **119**(5 Suppl 1): p. S17-23.

26. Maia-Fernandes, T., et al. *Cardiovascular actions of adiponectin: pathophysiologic implications*. Portuguese Journal of Cardiology, 2008. **27** (11) p. 1431-49.

27. Harada N, et al. *AT(1) receptor blockers increase insulin-like growth factor-I production by stimulating sensory neurons in spontaneously hypertensive rats.* Translational Research, 2009. **154**(3); p. 142-52.

28. Kissebah, A.H., et al. *Relation of body fat distribution to metabolic complications of obesity.* J Clin Endocrinol Metab, 1982. **54**(2); p. 254-60.

29. Fujioka, S., et al. *Contribution of intra-abdominal fat accumulation to the impairment of glucose and lipid metabolism in human obesity*. Metab Clin Exp, 1987. **36**(1); p. 54-9.

30. Rong, X., et al. *An adipose tissue-independent insulin-sensitizing action of telmisartan: a study in lipodystrophic mice*. J Pharmacol Exp Ther, 2009. p. 1125.

31. Steinmetz, M., et al. *Synergistic effects of telmisartan and simvastatin on endothelial progenitor cells*. J Cell Mol Med, 2009. p. 1195.

32. Nakano, A., et al. *Telmisartan inhibits cytokine-induced nuclear factor-kappaB activation independently of the peroxisome proliferator-activated receptor-gamma*. Hypertens Res, 2009. **32**(9); p. 765-9.

33. Tian, Q., et al. *Inhibition of tumor necrosis factor-alpha-induced interleukin-6 expression by telmisartan through cross-talk of peroxisome proliferator-activated receptor-gamma with nuclear factor kappaB and CCAAT/enhancer-binding protein-beta*. Hypertension, 2009. **53**(5); p. 798-804.

34. Brillante, D., et al. Effects of cardiovascular angiotensin II type 1 receptor blockade on nitric oxide synthase inhibition in patients with insulin resistance syndrome. Blood Press, 2009; p. 1-7.

35. Sugimoto, K., et al., *Telmisartan but not valsartan increases caloric expenditure and protects against weight gain and hepatic steatosis.* Hypertension, 2006. **47**(5): p. 1003-9.

36. Makita, S., et al. *Effects of telmisartan on adiponectin levels and body weight in hypertensive patients with glucose intolerance*. Metab Clin Exp, 2008. **57**(10); p. 1473-8.

37. Aubert, G., et al. Neuroendocrine characterization and anorexigenic effects of telmisartan in diet- and glitazone-induced weight gain. Metab Clin Exp, 2009. p. 1200.

38. Derosa, G., et al. *Telmisartan and irbesartan therapy in type 2 diabetic patients treated with rosiglitazone: effects on insulin-resistance, leptin and tumor necrosis factor-alpha.* Hypertens Res, 2006. **29**(11); p. 849-56.

39. Okuno, A., et al. *Troglitazone increases the number of small adipocytes without the change of white adipose tissue mass in obese Zucker rats*. J Clin Invest, 1998. **101**(6); p. 1354-61.

40. Miyazaki, Y., et al. *Effect of pioglitazone on abdominal fat distribution and insulin sensitivity in type 2 diabetic patients*. J Clin Endocrinol Metab, 2002. **87**(6); p. 2784-91.

41. Laplante, M., et al. *Mechanisms of the depot specificity of peroxisome proliferator-activated receptor gamma action on adipose tissue metabolism*. Diabetes, 2006. **55**(10); p. 2771-8.

42. Makino, H., et al., *Microalbuminuria reduction with telmisartan in normotensive and hypertensive Japanese patients with type 2 diabetes: a post-hoc analysis of The Incipient to Overt: Angiotensin II Blocker, Telmisartan, Investigation on Type 2 Diabetic Nephropathy (INNOVATION) study*. Hypertens Res, 2008. **31**(4): p. 657-64.

43. Tagami, T., et al. *A selective peroxisome proliferator-activated receptor-gamma modulator, telmisartan, binds to the receptor in a different fashion from thiazolidinediones*. Endocrinology, 2009. **150**(2); p. 862-70.

**APPENDIX I**

**Data and Safety Monitoring Board Members**-TBD

**APPENDIX II**

**UCLA CARE CENTER**
**CONSENT TO PARTICIPATE IN RESEARCH**

**Metabolic Abnormalities, Telmisartan, and HIV Infection Trial**

**Lay Language Title: The MATH Trial**

## INTRODUCTION

You are being invited to participate in a research study because you are receiving treatment for HIV infection and have increased abdominal fat. The following information is provided to help you make an informed decision about whether or not to participate in this study. This is a consent form. It gives you information about this study. You should review this information and ask questions about anything you do not understand. The study staff will talk with you about this information. You are free to ask questions about this study at any time. If you agree to take part in this study, you will be asked to sign this consent form. You will get a signed and dated copy to keep. Your participation in this study is voluntary. You may decide not to take part or to withdraw from the study at any time without losing the benefits to your routine medical care.

This is a research study to see whether fat accumulation either under the skin or in the body's organs (for example, the liver) improves in men and women who take telmisartan. We will be looking at how the amount of fat in the body changes when HIV-positive persons on effective antiretroviral therapy take telmisartan. We will be using a CT scan to make this comparison. Telmisartan is not an HIV medication. It is a medication designed to treat blood pressure, but has been shown to improve fat gain in the organs in people both with and without high blood pressure. The study drug is made by Boehringer Ingelheim, and this trial is sponsored by the California HIV/AIDS Research Program (CHRP). More information on telmisartan is provided below.

If you do not receive your HIV care at the UCLA CARE Center, it is important that your primary HIV care provider know that you are taking part in this study. Your study doctor will communicate with your primary HIV care provider to ensure coordination of your HIV care during your participation in this study.

Your participation in this study involves 8 visits over a period of about 24 weeks. This study will involve approximately 35 people.

## DISCLOSURE

Your health care provider may be an investigator for this study, and as an investigator, is interested both in your clinical welfare and the conduct of this study. Before entering this study or at any time during the research, you may ask for a second opinion about your care from another doctor who is in no way associated with this project. You are not under any obligation to participate in any research project offered by your doctor. The study doctor will not be paid by Boehringer Ingelheim for conducting this study.

The study is sponsored by CHRP. The Principal Investigator of this study, Dr. Jordan Lake, has not received any other payments from CHRP for services provided.

**PROCEDURES**

There are several procedures that you will be asked to undergo if you agree to take part in the study and sign this consent form. These are described below and in a chart at the end of this form. **All procedures are being done for research purposes only.**

The study will consist of 8 visits to the study doctor over the period of approximately 24 weeks.

**Visit 1 - Screening**

After obtaining informed consent, a series of baseline evaluations will be performed.

During this visit, you will be asked to provide us with information, to the best of your ability, about your medical history and some personal information. The study doctor will ask about things such as: whether or not you smoke tobacco or drink alcohol, whether any illnesses run in your family, what illnesses (including HIV-related illness) and surgeries you have had, and which medications you take. We will also ask for your permission to let us contact your HIV doctor to find out more information about your health, including the results of your most recent HIV viral load test. The research staff must obtain this information to determine whether or not you are eligible to participate in the study.

Measurements of your hips and waist, will be collected. The research staff will complete a physical exam, take your height, weight, vital signs (blood pressure, pulse, temperature, breathing rate), and take some blood to send to the laboratory for testing. About 4 teaspoons of blood will be taken to test for the following:

- safety labs (liver enzymes, blood chemistry, and blood counts)
- HIV viral load

For women capable of having children, a urine or blood pregnancy test will be performed as part of the safety lab evaluation at each visit. You must notify the research staff if you are pregnant, think you may be pregnant, or if you are trying to become pregnant.

**Visit 2 Baseline (within 30 days of screening)**

The research staff will review all the information from your previous visit and your medical records to ensure you are still able to participate in the study. The research staff will ask you about any medicines you are taking, and if you have been sick since your last visit. **You will be asked to come in fasting [not having anything to eat or drink (except water and medications) for at least 8 hours].** Your vital signs (blood pressure, temperature, breathing rate, and pulse) will be taken, and you will be weighed. If you have any complaints or physical concerns, a physical exam will also be performed. A urine sample and about 2 tablespoons of blood will be taken to test for the following:

- safety labs
- HIV viral load

- immune function
- fasting lipids (cholesterol, HDL, LDL, and triglyceride measurements) and insulin
- inflammatory markers

Measurements of your hips and waist will be collected.

To complete this visit, you will also be asked to undergo a CT scan. A CT scan (computerized tomography) is a picture of a cross section of your body taken by a machine that uses advanced x-ray technology. In this study, the CT scanner will take an image of your abdomen (at about the level of your belly button) in order to measure the amount of fat under your skin and in your organs. You will go to UCLA to have the CT scan performed, and we will help arrange this for you. A CT scan will be also required at week 24.

At this visit you will start taking telmisartan. After you receive your first bottle of telmisartan, always bring this medicine with you to your study visits.

**Visit 3 – Week 1**
During this visit you will have a targeted physical exam with weight and blood pressure measurement. This visit is primarily to make sure you are tolerating the new medicine well. No blood will be drawn at this visit.

**Visit 4 – Week 2**
During this visit you will have a targeted physical exam with weight and blood pressure measurement. This visit is primarily to make sure you are tolerating the new medicine well. No blood will be drawn at this visit.

**Visit 5 – Week 6**
During this visit you will have a targeted physical exam with weight and blood pressure measurement. A urine and blood sample will also be collected for safety labs. For these tests, approximately 2 teaspoons of blood will be drawn.

**Visit 6 – Week 12**
During this visit you will have a targeted physical exam with weight and blood pressure measurement. **You will be asked to come in fasting [not having anything to eat or drink (except water and medications) for at least 8 hours].** A urine sample and about 2 tablespoons of blood will be taken to test for the following:

- safety labs
- fasting lipids (cholesterol, HDL, LDL, and triglyceride measurements) and insulin
- inflammatory markers

**Visit 7 - Week 18**
During this visit you will have a targeted physical exam with weight and blood pressure measurement. A urine and blood sample will also be collected for safety labs. For these tests, approximately 2 teaspoons of blood will be drawn.

**Visit 8 – Week 24**
During this visit you will have a targeted physical exam with weight and blood pressure measurement. Your waist and hips will be measured again. **You will be asked to come in fasting [not having anything to eat or drink (except water and medications) for at least 8 hours].** A urine sample and approximately 2 tablespoons of blood will be collected to test for:

- safety labs
- HIV viral load
- immune function
- fasting lipids (cholesterol, HDL, LDL, and triglyceride measurements) and insulin
- inflammatory markers

To complete this visit, you will also be asked to undergo another CT scan within 14 days. You will go to UCLA to have the CT scan performed, and we will help arrange this for you.

Once you have completed the Week 24 visit, the study is over. You and your study doctor will discuss whether or not you would like to keep taking telmisartan. If you would like to continue taking telmisartan, your primary HIV doctor can prescribe it for you, but we will no longer be able to provide it to you.

**Premature Study Treatment/Background ART Discontinuation Evaluations**
If you discontinue telmisartan due to an adverse event, you will be followed to make sure your event is resolved. If you discontinue for any reason we would like for you to complete the week 12 and 24 evaluations, as listed in the Schedule of Events, before you are taken off study.

All samples will be destroyed at the end of this study.

## POTENTIAL RISKS AND DISCOMFORTS
Side effects are unpleasant feelings or conditions that people get when they take a medication. Most medications cause some side effects. Many of these side effects do not happen very often, and not every person who takes a drug will experience side effects. In fact, you may have no side effects from telmisartan at all. Even so, you should know what to expect from the medication that will be used in this study.

Any side effects, including changes in medical conditions you had prior to starting the study, should be reported to the study doctor. In addition, if you need to take any new medications or change the dose of a current medication during the study, you should report this to the study doctor.

It is important for your safety that you are completely truthful with the study staff about your medical history, how you feel, and the medications you are taking.

**RISKS OF TELMISARTAN**
Telmisartan has been studied in thousands of patients worldwide, but has never been studied specifically in HIV-positive patients. Telmisartan is believed to be compatible with most HIV medications. If you take the HIV medication maraviroc, your dose might need to be adjusted while on telmisartan. If this is the case, we will let your primary HIV doctor know. Because of the way telmisartan works in the body, there is the possibility of an interaction with etravirine and/or

nelfinavir, although none have been reported. However, to ensure your safety, you will not be allowed to participate in the study if you are currently taking etravirine (Intelence™) or nelfinavir (Viracept™).

**Studies is Subjects with High Blood Pressure**

In 6 major studies of patients with high blood pressure, telmisartan decreased systolic blood pressure (the “top” number in blood pressure) by 6-13mm Hg and diastolic blood pressure (the “bottom” number) by 6-8mm Hg. These changes in blood pressure occurred when telmisartan was given in doses ranging from 20-160mg daily. The maximum effect of telmisartan on blood pressure was at a dose of 80mg daily, and was less in African American than White patients. The effect of telmisartan on blood pressure did not vary by age, gender, or weight.

Telmisartan tablets have been evaluated for safety in more than 3700 patients with high blood pressure. The frequency and severity of side effects was generally low, most did not last long, and those that did occur were rarely severe enough to cause a person to stop taking telmisartan. In controlled trials, the overall incidence of adverse events was similar to placebo (sugar pill).

Side effects occurring in more than 1 out of a 100 patients treated with telmisartan AND more often than in patients treated with placebo are listed below. These events may or may not have been caused by telmisartan, but did occur more frequently in people taking telmisartan.

- Upper respiratory infection
- Back pain
- Sinus inflammation
- Diarrhea
- Sore throat

Side effects that occurred in 1 out of 100 people AND were equally as common as in patients taking placebo were:

- flu-like symptoms
- upset stomach
- muscle pain or soreness
- urine infection
- abdominal pain
- headache
- dizziness
- pain
- feeling tired
- coughing
- high blood pressure
- chest pain
- nausea
- swelling of the hands and/or feet.

1 case of angioedema (swelling of body tissue) occurred in the 3781 subjects treated with telmisartan.

Feeling lightheaded or faint because of decreased blood pressure was reported in 4 out of 1000 telmisartan-treated subjects.

Overall, only 41 of 1455 patients on telmisartan and 23 out of 380 patients taking placebo had to stop treatment because of side effects. Side effects did not occur more commonly at higher doses of telmisartan, were equally common in men and women, and did not vary by age or race/ethnicity.

Abnormal blood tests were rarely seen in patients taking telmisartan. Those that did occur were:

- Anemia (low red blood counts) was seen in 8 out of 1000 telmisartan patients compared with 3 out of 1000 placebo patients. No patients stopped telmisartan due to anemia.
- Kidney function: A significant worsening of kidney function was seen in 4 out 1000 telmisartan patients and 3 out of 1000 placebo patients. Only 1 telmisartan-treated patient stopped taking the medication due to worsening kidney function.
- Liver function: Occasional liver inflammation was seen in patients taking telmisartan, however, severe liver inflammation was seen only in patients taking placebo. No telmisartan-treated patients stopped taking the medication because of abnormal liver function.

**Studies in Patients with Normal Blood Pressure**

Telmisartan causes only small decreases in blood pressure, and has been given to people with normal blood pressure to help with problems such as: kidney function, high cholesterol, and fat gain in the organs of the body.

In a trial of patients with diabetes, telmisartan was shown to be safe and well-tolerated in patients with normal blood pressure. There was no difference in side effects between people who did and did not have high blood pressure.

**RISKS OF CT SCAN**

We are exposed to radiation on a daily basis both from natural (sun and earth) and human-made sources. In addition to the radiation that you may be exposed to as part of your medical care, you will receive two CT scans while participating in this research study.

The estimated radiation dose that you will receive as a result of the additional two CT scans is 1500 millirem, or 30% of the 5,000 limit allowed that radiation workers can be exposed to every year.

**RISKS OF BLOOD DRAWING**

Taking blood may cause pain, discomfort, bleeding, or bruising where the needle enters the body and, in rare cases, fainting, infection, or blood clots.

**RISKS OF FASTING**

You may experience lightheadedness or nausea from fasting.

In addition, there may be discomforts or risks to you which are presently not foreseeable.

**ANTICIPATED BENEFITS TO SUBJECTS**
During this study, your condition will be monitored closely. However, you may receive no benefit by participating in this study.

**ANTICIPATED BENEFITS TO SOCIETY**
Information gained about the safety and effectiveness of telmisartan in HIV-infected men and women may benefit patients in the future.

**ALTERNATIVES TO PARTICIPATION**
If you decide not to participate in this study, other treatments are available that may benefit you. You should discuss these options with your primary doctor. Alternatives to participation include both these treatments, and enrollment in other experimental protocols. There are no FDA-approved medications for the treatment of lipohypertrophy in HIV patients. Telmisartan is FDA-approved to treat high blood pressure and may be available from your health care provider outside of this study. Additionally, you may decide not to receive any treatment for your fat accumulation at this time.

**PAYMENT FOR PARTICIPATION**
For your time, and to help reimburse you for your expenses while you are on the study, you will receive $25 for each clinic visit. Additionally, parking vouchers are available for the days when you are required to go to UCLA Medical Center for a CT scan. Parking at the UCLA CARE Center is free of charge. Bus tokens are available to all bus riders without a bus pass in an amount equivalent to the round-trip cost of coming from your home or work to the UCLA CARE Center and/or UCLA Medical Center.

If you decide to leave the study or if you are taken off study for any reason, you will be reimbursed for all study days you have participated in. Total possible payment for your completion of the entire study is $200.

**FINANCIAL OBLIGATION**
There will be no cost to you for the study drug (telmisartan), clinic visits, examinations, or laboratory and test procedures that are part of this study. You or your insurance provider should continue to pay for expenses for your current medical care and prescriptions, including your HIV medications.

It is possible that your insurance will not pay for all the treatments and tests you will receive if you participate in this research. That is because many insurance companies, HMOs, and health benefits plans do not cover the cost of standard treatments that are provided as part of a research study. If that happens, you will be responsible for all charges related to your treatment including cost of the medication, pharmacy dispensing fees, office visits, laboratory charges, radiological studies, and hospitalization charges (if necessary). The study doctor will provide you with an estimate of the cost of participation in the research, if applicable.

**EMERGENCY CARE AND COMPENSATION FOR INJURY**
If you are injured as a direct result of research procedures, you will receive treatment at no cost. The University of California and CHRP do not provide any other form of compensation for injury.

## PRIVACY AND CONFIDENTIALITY

The only people who will know that you are a research subject are members of the research team and, if appropriate, your physicians and nurses. No information about you, or provided by you during the research, will be disclosed to others without your written permission, except if necessary to protect your rights or welfare (for example, if you are injured and need emergency care) or if required by law. People who may review your records include: CHRP, the UCLA Office for Protection of Research Subjects, and their designees. As a result, they may see your name, but they are bound by the rules of confidentiality not to reveal your identity to others.

When the results of the research are published or discussed in conferences, no information will be included that would reveal your identity.

Authorized representatives of the Food and Drug Administration (FDA), the study sponsor and those working for the sponsor, independent ethics committees, and inspectors from foreign government regulatory agencies may need to review records of individual subjects. However, no representative may take away any identifying information about you from the clinic site (e.g., names, addresses). As a result, they may see your name, but they are bound by rules of confidentiality not to reveal your identity to others.

There is a second, more detailed explanation of how health data about you will be used and shared with others that is required by federal law, i.e., Health Insurance Portability and Accountability Act. This consent is a separate form. If you decide not to sign this separate form, you will not be allowed to participate in this research study.

## PARTICIPATION AND WITHDRAWAL

Your participation in this research is VOLUNTARY. If you choose not to participate, that will not affect your relationship with UCLA (or UCLA Medical Center) or your right to health care or other services to which you are otherwise entitled. If you decide to participate, you are free to withdraw your consent and discontinue participation at any time without prejudice to future care at UCLA.

## WITHDRAWAL OF PARTICIPATION BY THE INVESTIGATOR

Your participation in this study may be stopped at any time without you being asked. Your participation in the study may be stopped for any of the following reasons:

- The investigator decides it is in the best interest of your health and welfare
- You are unable to follow the investigator’s instructions
- If the sponsor, the FDA, or UCLA stops the study
- Administrative reasons
- If you become pregnant
- If your health changes or if you develop a medical problem that disqualifies you from participating in the study (for example, if your viral load goes up).
- If you have to start taking any medications that are not allowed for patients in the study.

## NEW FINDINGS

During the course of the study, you will be informed of any significant new findings, good or bad, such as changes in the risks or benefits to participating in the research study, or new alternatives to participation that might cause you to change your mind about continuing in the study. If new

information is provided to you, your consent to continue participating in this study will be re-obtained.

**IDENTIFICATION OF INVESTIGATORS**

In the event of a research related injury or if you experience an adverse reaction, please immediately contact one of the investigators listed below. If you have any questions about the research, please feel free to contact:
[Names of study faculty/staff]

**RIGHTS OF RESEARCH SUBJECTS**

You may withdraw your consent at any time and stop participating in the study without penalty. You are not waiving any legal claims, rights, or remedies because of your participation in this research study. If you have questions regarding your rights as a research subject, you may contact the Office for Protection of Research Subjects, [address and phone number].

**SIGNATURE OF RESEARCH SUBJECT**

I have read the information provided in this consent form. I have been given an opportunity to ask questions, and all of my questions have been answered to my satisfaction. I have been given a copy of the Subject's Bill of Rights, and I will be given a signed and dated copy of this consent form after the study physician and I sign it.

**BY SIGNING THIS FORM, I WILLINGLY AGREE TO PARTICIPATE IN THE RESEARCH IT DESCRIBES.**

________________________________________

Name of Subject

________________________________________ _______________

Signature of Subject Date

**SIGNATURE OF INVESTIGATOR**

I have explained the research to the subject and answered all of his/her questions. I believe that he/she understands the information described in this document and freely consents to participate.

________________________________________

Name of Investigator

________________________________________ ______________________________

Signature of Investigator Date (must be the same as subject)

## CHART OF PROCEDURES

| | Screen | Baseline* | Week 1 | Week 2 | Week 6 | Week 12* | Week 18 | Week 24* |
|---|---|---|---|---|---|---|---|---|
| Informed Consent, Patient's Bill of Rights, HIPPA, medical information release | X | | | | | | | |
| Targeted physical exam with weight, blood pressure | | X | X | X | X | X | X | X |
| Complete physical exam and height | X | | | | | | | |
| CT- L4/5 | | X | | | | | | X |
| Mid-waist, hip and neck circumferences and waist/hip ratio | X | X | | | | | | X |
| HIV-1 RNA | X | X | | | | | | X |
| Safety labs | X | X | | | X | X | X | X |
| Pregnancy Test** | X | X | | | X | X | X | X |
| CD4 cell counts | | X | | | | | | X |
| Fasting lipids, hsCRP, banked serum and plasma | | X | | | | X | | X |
| Urine samples for markers of oxidant stress and endothelial activation, protein | | X | | | | X | | X |

*Fasting Visit: please do not eat or drink for at least 8 hours prior to your visit (except for water and medications)

** If you are a woman who is able to become pregnant.

## APPENDIX III – CLINICAL TRIALS PROTOCOL REGISTRATION #
